# Finding Statistically Significant Communities in Networks

**DOI:** 10.1371/journal.pone.0018961

**Published:** 2011-04-29

**Authors:** Andrea Lancichinetti, Filippo Radicchi, José J. Ramasco, Santo Fortunato

**Affiliations:** 1 Complex Networks and Systems Lagrange Laboratory, Institute for Scientific Interchange (ISI), Torino, Italy; 2 Physics Department, Politecnico di Torino, Torino, Italy; 3 Howard Hughes Medical Institute (HHMI), Northwestern University, Evanston, Illinois, United States of America; 4 Instituto de Física Interdisciplinar y Sistemas Complejos IFISC (CSIC-UIB), Palma de Mallorca, Spain; Tel Aviv University, Israel

## Abstract

Community structure is one of the main structural features of networks, revealing both their internal organization and the similarity of their elementary units. Despite the large variety of methods proposed to detect communities in graphs, there is a big need for multi-purpose techniques, able to handle different types of datasets and the subtleties of community structure. In this paper we present OSLOM (Order Statistics Local Optimization Method), the first method capable to detect clusters in networks accounting for edge directions, edge weights, overlapping communities, hierarchies and community dynamics. It is based on the local optimization of a fitness function expressing the statistical significance of clusters with respect to random fluctuations, which is estimated with tools of Extreme and Order Statistics. OSLOM can be used alone or as a refinement procedure of partitions/covers delivered by other techniques. We have also implemented sequential algorithms combining OSLOM with other fast techniques, so that the community structure of very large networks can be uncovered. Our method has a comparable performance as the best existing algorithms on artificial benchmark graphs. Several applications on real networks are shown as well. OSLOM is implemented in a freely available software (http://www.oslom.org), and we believe it will be a valuable tool in the analysis of networks.

## Introduction

The analysis and modeling of networked datasets are probably the hottest research topics within the modern science of complex systems [1]–[7]. The main reason is that, despite its simplicity, the network representation can disclose some relevant features of the system at large, involving its structure, its function, as well as the interplay between structure and function. The elementary units of the system are reduced to simple points, called *vertices* (or *nodes*), while their pairwise relationships/interactions are pictured as *edges* (or *links*). It is fairly easy to spot the two main ingredients of a graph in many instances. Therefore networks can be found everywhere: in biology (e. g., proteins and their interactions), ecology (e. g., species and their trophic interactions), society (e. g., people and their acquaintanceships). Other noteworthy examples include the Internet (routers/autonomous systems and their physical and/or wireless connections), the World Wide Web (URLs and their hyperlinks), etc.

The structure of most networks, beneath the intrinsic disorder due to the stochastic character of their generation mechanisms, reveals a high degree of organization. In particular, vertices with similar properties or function have a higher chance to be linked to each other than random pairs of vertices and tend to form highly cohesive subgraphs, which are called *communities* (also *modules* or *clusters*). Examples of communities are groups of mutual acquaintances in social networks [8]–[10], subsets of Web pages on the same subject [11], compartments in food webs [12], [13], functional modules in protein interaction networks [14], biochemical pathways in metabolic networks [15], [16], etc.

Detecting communities in graphs may help to identify functional subunits of the system and to uncover similarities among vertices that are not apparent in the absence of detailed (non-topological) information. Vertices belonging to the same community may be classified according to their structural position within the cluster, which may be correlated to their role. Vertices in the core of the cluster may have a function of control and stability within the module, whereas boundary vertices are likely to be mediators between different parts of the graph. The community structure of a network can also be a powerful visual representation of the system: instead of visualizing all the vertices and edges of the network (which is impossible on large systems), one could display its communities and their mutual connections, obtaining a far more compact and understandable description of the graph as a whole. It is thus not surprising that community detection in graphs has been so extensively investigated over the last few years [17]. A huge variety of different methods have been designed by a truly interdisciplinary community of scholars, including physicists, computer scientists, mathematicians, biologists, engineers and social scientists.

However, most algorithms currently available cannot handle important network features. Many methods are designed to find clusters in undirected graphs, and cannot be easily (or not at all) extended to directed graphs. However, there are many datasets for which edge directedness is an essential feature. Citation networks, food webs and the Web graph are but a few examples. Similar problems arise when edges carry weights, indicating the strength of the interaction/affinity between vertices, although extensions are generally easier in this case.

Likewise, the great majority of algorithms are not capable to deal with the peculiar features of community structure. For example, each vertex is typically assigned to a single cluster, while in several instances, like in social networks, vertices are typically shared between two or more clusters. In such cases communities are *overlapping* (and partitions become *covers*) and very few methods account for this possibility [18]–[25], which considerably increases the complexity of the problem. Furthermore, community structure is very often *hierarchical*, i.e. it consists of communities which include (or are included by) other communities. Hierarchies are common in human societies and are crucial for an efficient management of large organizations. Simon pointed out that hierarchy gives robustness and stability to complex systems, yielding an evolutionary advantage on the long run [26]. However, most community finding methods typically look for the “best” partition of a network, disregarding the possible existence of hierarchical structure. Instead, a method should be able to recognize if there is hierarchical structure and, if yes, identify the corresponding levels [27]–[29].

It is also very important for a method to distinguish communities from pseudo-communities. The existence of clusters indicate a preference by some groups of vertices to link to each other. But, if the linking probability is the same for all pairs of vertices, like in random graphs, no communities are expected. In this case, concentrations of edges within groups of vertices are simply the result of random fluctuations, they do not represent potentially non-trivial structures. Many algorithms are not able to see this difference and find clusters in random graphs as well, although they are not meaningful. Scholars have just begun to assess the issue of significance of clusters [30], [31].

Finally, given the recent availability of time-stamped networked datasets, it is now possible to carry out quantitative studies on the dynamics of community structure, about which very little is known [32]–[37]. A simple way to treat dynamic datasets is to analyze snapshots of the system at different times separately, and then map communities of different snapshots onto each other, such that one can follow the dynamic of each cluster in time. However, focusing on individual snapshots means disregarding the information on the system at previous times. Ideally a partition/cover of the system at time 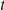 should be faithful both to its structure at time 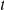 and to its history [34], [37].

In this paper we propose the first method able to meet all requirements listed above, the Order Statistics Local Optimization Method (OSLOM). It is a method that optimizes locally the statistical significance of clusters, defined with respect to a global null model. The concept of statistical significance is inspired by recent work of some of the authors [31], [38]. The paper is structured as follows. After introducing the method, we test its performance on artificial benchmark graphs, comparing it with the performances of the best algorithms currently available. Next, we pass to the analysis of real networks, followed by a final discussion on the work. Some of the tests on artificial and real networks are reported in the Supporting Information S1.

## Methods

### Statistical significance of clusters

In this section we explain how to estimate the statistical significance of a given cluster. OSLOM will use the significance as a fitness measure in order to evaluate the clusters. Following our previous work [31], we define it as the probability of finding the cluster in a random null model, i. e. in a class of graphs without community structure. We choose the configuration model [39] as our null model. This is a model designed to build random networks with a given distribution of the number of neighbors of a vertex (degree). The networks are generated by joining randomly vertices under the constraint that each vertex has a fixed number of neighbors, taken from the pre-assigned degree distribution. This is basically the same null model adopted by Newman and Girvan to define modularity [40].

**Figure 1:**
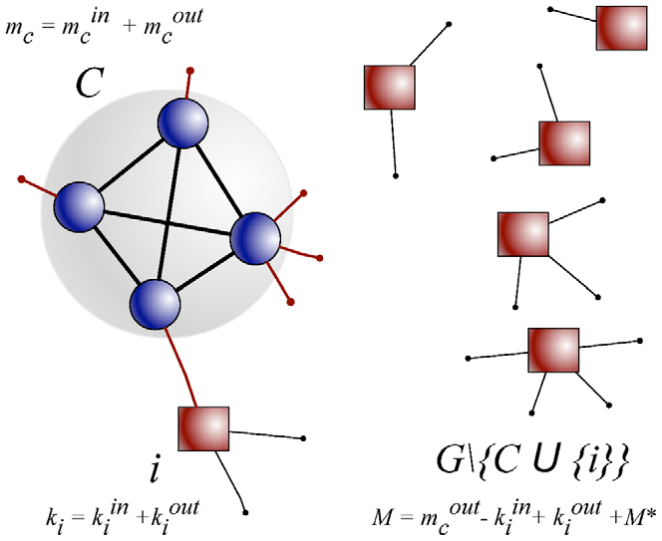
A schematic representation of a subgraph 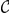, whose significance is to be assessed. The subgraph 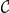 is embedded within a random graph generated by the configuration model. The degrees of all vertices of the network are fixed, in the figure we have highlighted the degrees of 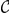 (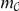), of the vertex 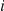 at the center of the analysis (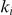) and of the rest of the graph 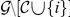 (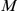). These quantities are expressed as sums of contributions which are internal to their own set of vertices (as 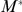) or related to subgraph 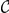 (in or out). This notation is used in the distribution of Eq. 1.

We start from a graph 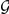 with 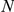 vertices and 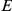 edges. The framework for the analysis is sketched in Fig. 1. We are given a subgraph 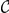, whose significance is to be assessed, a vertex 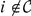 and the degree of the vertices of the rest of the graph 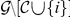. The degree of subgraph 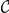 is 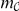, 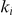 is the degree of 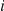, and the rest of vertices have a total degree 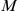. We can separate the above quantities in the contributions internal or external to 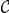 (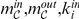 and 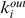); the internal degree of 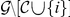 is 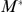 (Fig. 1).

Let us suppose that 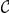 is a subgraph of graphs generated by the configuration model, where each vertex maintains the degree it has on the graph 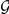 at study. We assume that the internal degree 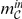 of the subgraph is fixed. If all the other edges of the network are randomly drawn, the probability that 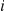 has 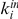 neighbors in 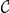 can be written as [38]

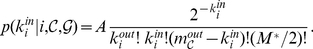
(1)This equation enumerates the possible configurations of the network with 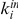 connections between 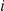 and 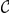. The factorials of the formula express the multiplicity of configurations with fixed values of 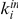, 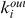, 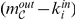 and 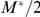, whereas the power of 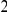 in the numerator stays for the multiplicity coming from the permutation of the extremes of edges lying between 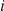 and 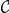. Several of the terms in the expression can actually be written as a function of constants and 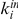, such as 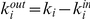 and 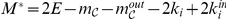. The normalization factor 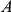 includes terms not depending on 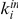 and ensures that
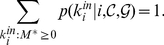
(2)Further details on the numerical implementation of the formula in Eq. 1, as well as on the different approximations taken and their limits, are included in the Supporting Information S1.

The probability of Eq. 1 provides a tool to rank the vertices external to 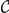 according to the likelihood of their topological relation with the group. If vertex 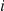 shares many more edges with the vertices of subgraph 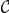 than expected in the null model, we could consider the inclusion of 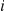 in 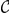, since the relationship between 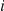 and 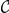 is “unexpectedly” strong. In order to perform the ranking the cumulative probability 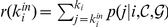 of having a number of internal connections equal or larger than 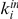 is estimated, following Ref. [31]. Given that the vertex degree is a discrete variable, the cumulative distribution has a specific step-wise profile for each value of 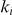. In order to facilitate the comparison of vertices with different degrees, we implement a bootstrap strategy by assigning to each vertex 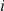 a value of 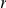, 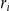, randomly drawn from the interval 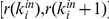. **This choice is important for a meaningful estimate of the clusters' significance; other options (e. g., taking the middle points of the interval) could lead to the identification of meaningful clusters in random graphs.** The bootstrap introduces a stochastic element in the assessment procedure, which will, in turn, lead to the use of Monte Carlo techniques.

**Figure 2:**
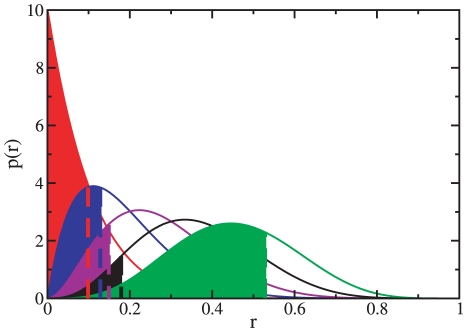
Probability distributions of the scores 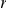 of vertices external to a given subgraph 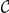 of the graph. The score 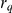 is the 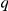-th smallest score of the external vertices. In this particular case there are 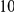 external vertices. In the figure, we plot 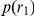, 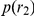, 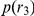, 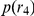, 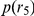 (from left to right). As an example, the shaded areas show the cumulative probability 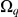 for a few values of 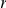 that would correspond to the values estimated in a practical situation. In this case, the black area, 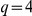, is the least extensive and so 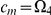. If 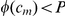, the vertices with scores 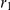, 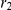, 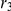 and 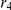 will be added to 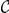.

The variable 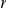 bears the information regarding the likelihood of the topological relation of each vertex with 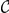 and has an important feature: it is a uniform random variable distributed between zero and one for vertices of our null model graphs. Calculating its order statistic distributions is thus a relatively easy task. The first candidate among the external vertices to be part of 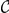 is the vertex with the lowest value of 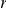, that we indicate 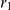. The cumulative distribution of 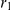 in the null model is then given by
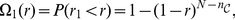
(3)where 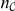 is the number of vertices in 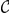. In general, let 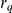 be the value of variable 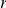 with rank 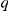 (in increasing order of the variable 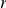). Its cumulative distribution is (Fig. 2):
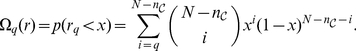
(4)


The reason for the use of order statistics is that we assume that clustering methods tend to include in each community those vertices which are most strongly connected to vertices of the community. Due to correlations (the vertices in the clusters tend to be connected), we cannot calculate the statistics of the internal connections to the clusters, but we can do it safely for the external vertices. The values of the different 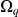 inform us of how much the external vertices of a group are compatible with the statistics expected in the null model. To evaluate the full group, we define 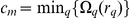 among all the neighbors of 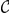, where 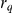 are their corresponding ranked values for the 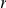 variable. The distribution of 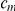 can be easily tabulated numerically since it only depends on 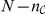. The cumulative distribution will be denoted as 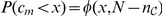. In the following, we call 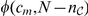 the *score* of the cluster 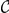.

### Single cluster analysis

**Figure 3:**
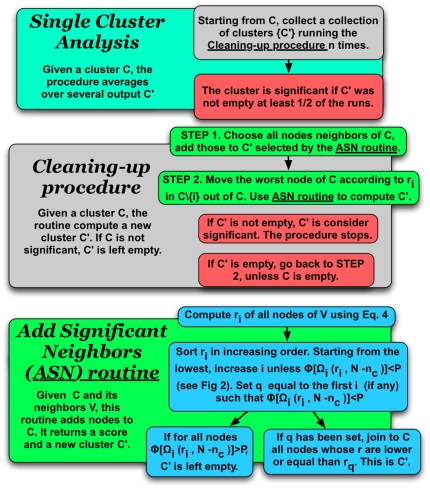
Schematic diagram of the single cluster analysis.

Now that a score to evaluate the statistical significance of the clusters has been introduced, the next step is to optimize the score across the network by dividing it into proper clusters. We describe first the optimization of a single cluster score and will extend later the method to deal with the full network. First of all one has to give the method a certain tolerance, in the following referred to as 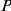. This parameter establishes when a given value of the score is considered significant. Our procedure consists of two phases: first, we explore the possibility of adding external vertices to the subgraph 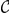; second, non-significant vertices in 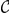 are pruned. They are described below and illustrated schematically in Fig. 3.

1. For each vertex 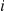 outside 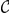 and connected to it by at least one edge the variable 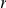 is computed. Then we calculate 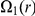 for the vertex with the smallest 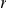, by using Eq. 3. If 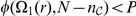, we add the corresponding vertex to the subgraph, which we now call 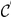. If 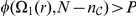, one checks the second best vertex, the third best vertex, etc. If there is finally a vertex, say the 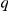-th best vertex, for which 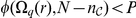, one includes all 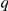 best vertices into subgraph 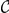, yielding subgraph 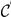. At this point, no other vertex outside 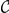 deserves to enter the community since all the external vertices are compatible with the statistics of the random configuration model. It may also happen that the inequality 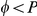 above holds for no external vertex, in which case we add no vertices to 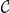 and 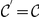. Either way, we pass to the second stage with the subgraph 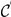.
2. For each vertex 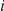 in 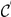 the variable 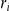 with respect to the set 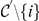 is estimated. We pick the “worst” vertex 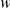 of the cluster, i. e. the vertex with the highest value of 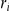. To check for its significance we repeat step 1 for the subgraph 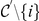. If 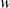 turns out to be significant, we keep it inside 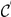 and the analysis of the cluster is completed. Otherwise, 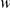 is moved out of 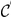 and one searches for the worst internal vertex of 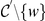. At some point we end up with a cluster 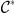, whose internal vertices are all significant and the process stops.

The two-steps procedure is a way to “clean up” 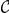. A cluster is left unchanged only if all the external vertices are compatible with the null model and all the internal vertices are not. A few remarks are important here:

- There can be both *good* vertices outside 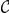 and *bad* ones inside. It is important to perform the complete procedure described above, which guarantees that the final cluster is significant with respect to the present null model (see also Ref. [31]).
- The procedure is not deterministic, because of the stochastic component in the computation of the cumulative probability 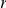. So one shall repeat all the steps several times. The cluster analysis may deliver a subgraph 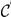, in general different from 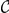, or an empty subgraph. For each vertex 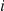 we compute the participation frequency 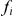, defined as the ratio between the number of times 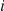 belongs to any non-empty 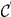 and the total number of iterations leading to non-empty subgraphs. In general, we consider the subgraph 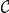 to be a significant cluster if the single cluster analysis yields a non-empty subgraph 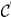 in more than 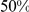 iterations. The final “cleaned” cluster includes those vertices for which 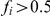.
- In the worst-case scenario, the complexity of the cluster analysis scales with the number of vertices of 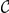, times the number of neighbors of 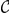, times the number of loops needed to have reliable values for the 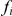
's. The situation can be considerably improved by keeping track of the order of the external vertices at each step (using suitable data structures) and by computing the score only for some reasonably good vertices. For instance, one could pick just those vertices with 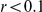. We numerically checked that changing this threshold does not affect the results, but leads to a faster algorithm.

### Network analysis

The previous procedure deals with a single cluster 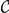. It finds the external significant vertices and includes them into 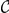. It also prunes those internal vertices that are not statistically relevant. Now we extend this procedure by introducing an algorithm able to analyze the full network. In order to do so, we follow the method proposed by some of the authors in Ref. [23]. The starting point is a single vertex, taken at random, in the absence of any information. Let us suppose that we start from a random vertex 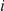 and that our first group is 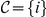. The method proceeds as follows:

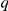
 vertices are added to 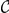, considering the most significant among the neighbors of the cluster. The number 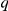 is taken from a distribution, which in principle can be arbitrary. We choose a power law with exponent 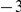.
2. Perform the single cluster analysis.

We repeat the whole procedure starting from several vertices in order to explore different regions of the network. This yields a final set of clusters that may overlap. Such type of local optimization was originally implemented in the Local Fitness Method [23], to handle overlapping communities. The algorithm stops when it keeps finding *similar* modules over and over. Ideally one wishes to encounter the exact same clusters repeatedly. However, the stochastic element introduced when calculating the vertex score can lead vertices, whose score is close to the threshold, to change their group assignments from one realization to another. This can be a problem when we are trying to decide whether two groups in different instances correspond to the same cluster. As a practical rule, we say that two groups 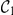 and 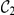 are similar if 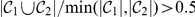, in which case they deserve further attention. Indeed, it turns out that many of the clusters found are very similar or combinations of each other. This leads to a very important question: given a set of significant clusters, which ones should be kept?

Let us consider the problem of choosing between two clusters 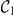 and 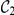 and the union of the two, 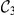. A solution is to consider the subgraph 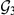 of the vertices in 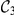 and see if 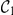 and 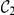 are significant as modules of 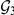. Strictly speaking we consider 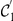 and 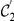 which are the cleaned up clusters within 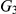 (i.e. with respect to subgraph 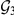 only, neglecting the rest of the network). We discard 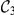 if 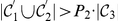, where we set 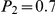. Otherwise we discard 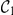 and 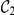 and we keep the union 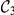. Instead, if we have to decide among a set of 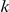 clusters and their union, the condition to prefer the submodules is 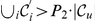.

In general, we check if each cluster has significant submodules, by looking for modules in the subgraph given by the cluster and using the condition above to decide which ones to take. This leads to a set of significant minimal clusters, where minimal means that they have no significant internal cluster structure, according to the condition above. We also need to check whether unions of such minimal clusters do have internal cluster structure, according to our rule, to decide whether the clusters have to be kept separated or merged. After doing this, we still end up with many *similar* modules. Given a pair of similar modules (in the sense defined above), we first check if their union has significant cluster structure: if it does not, we merge the two clusters, otherwise we systematically prefer the bigger one (if they are equal-sized, we pick the cluster with smaller score).

After the completion of this procedure, the output is a cover of the network. To reduce the stochasticity introduced by the bootstrap, the procedure is repeated in order to obtain several covers. All clusters of the covers are analyzed as described above to select among them the ones which will appear in the final output.

The parameter values may affect the outcome of OSLOM. The value of the significance level 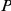 plays an important role for the determination of the size of the clusters found by OSLOM. In general, small values of 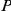 lead to the identification of large clusters, and large values of 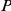 allow the identification of small clusters. Likewise, large values of the parameter 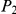, which controls the internal structure of modules, generally lead to the identification of large clusters. The influence of the parameter values is however relevant only when the community structure of the network is not pronounced. When modules are well defined, the results of OSLOM do not depend on the particular choice of the parameter values.

### OSLOM

**Figure 4:**
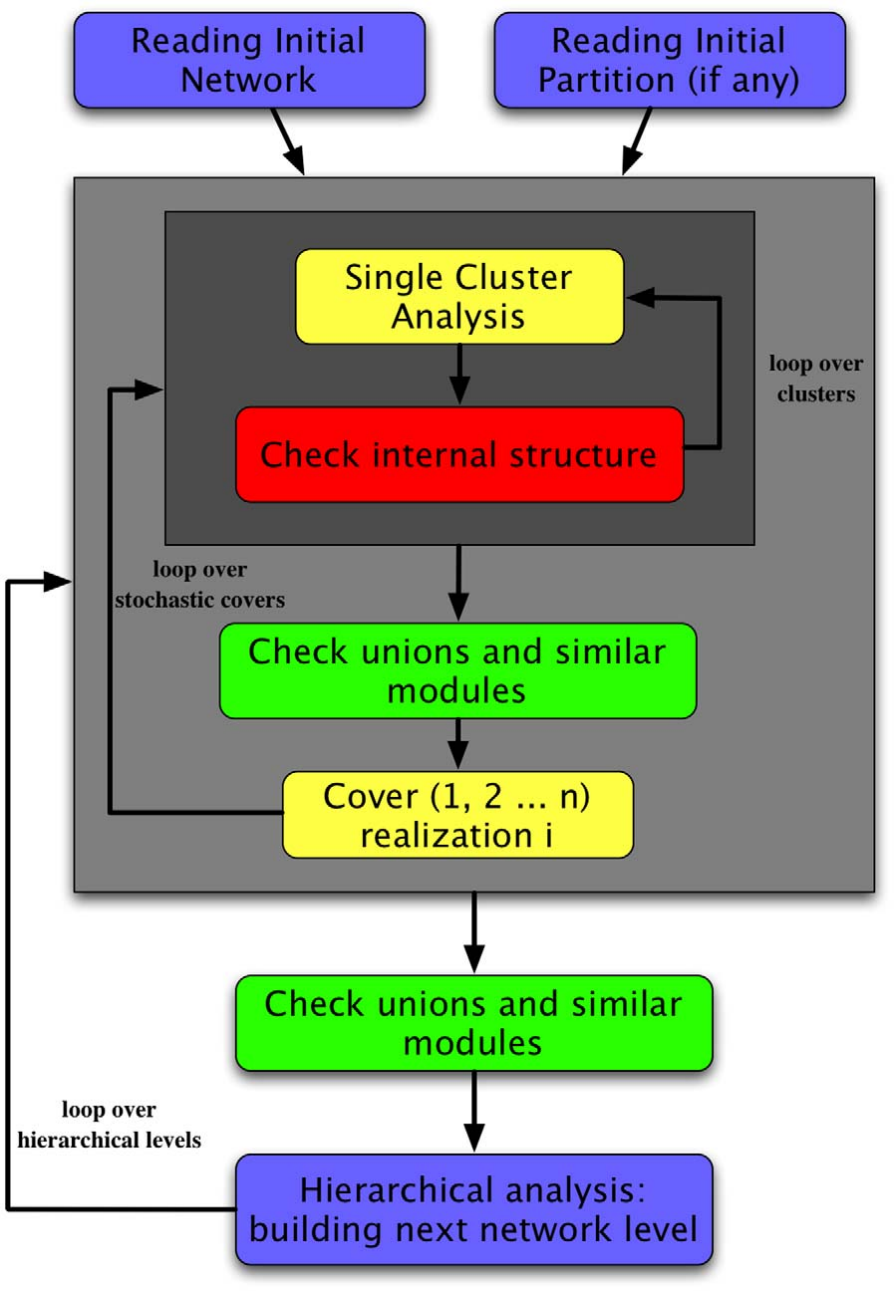
Flux diagram of OSLOM. The levels of grey of the squares represent different loop levels. One can provide an initial partition/cover as input, from which the algorithm starts operating, or no input, in which case the algorithm will build the clusters about individual vertices, chosen at random. OSLOM performs first a cleaning procedure of the clusters, followed by a check of their internal structure and by a decision on possible cluster unions. This is repeated with different choices of random numbers in order to obtain better statistics and a more reliable information. The final step is to generate a super-network for the next level of the hierarchical analysis.

We have described the cleaning of a single cluster and how the full network is analyzed. In the following, all the ingredients are assembled together to form the algorithm that we call OSLOM (Order Statistics Local Optimization Method). A flux diagram summarizing how it works can be seen in Fig. 4. OSLOM consists of three phases:

- First, it looks for significant clusters, until convergence;
- Second, it analyzes the resulting set of clusters, trying to detect their internal structure or possible unions thereof;
- Third, it detects the hierarchical structure of the clusters.

To speed up the method, one can start from a given partition/cover delivered by another (fast) algorithm or from *a priori* information. In those cases, the first step will be to clean up the given clusters.

Once the set of minimal significant clusters has been found, the analysis of the hierarchies consists of the following steps. We construct a new network formed by clusters, where each cluster is turned into a supervertex and there are edges between supervertices if the representative clusters are linked to each other. The resulting superedges are weighted by the number of edges between the initial clusters. There is the problem of properly assigning edges between clusters, if the edges are incident on overlapping vertices. Suppose to have an edge whose endvertices 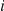 and 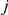 belong to 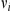 and 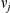 clusters, respectively. This edge lies simultaneously between any pair of clusters 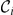 and 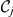, with 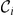 including 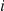 and 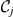 including 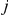. The contribution of the edge to the superedge between 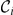 and 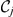 equals 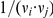. The resulting non-integer weights may lead to non-integer values for the weight of superedges, whereas we need integer values in order to use Eq. 1. For this reason, the weight of each superedge is rounded to the nearest integer value. We stress that the weight we deal with here indicates just how to “split” edges, it is not related to the weight that edges may carry. If the original network is weighted, the rescaled weight of an edge is 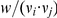, 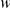 being the weight of the edge in the network. Once the supernetwork has been built, one applies the method again, obtaining the second hierarchical level. The latter is turned again into a supernetwork, as we explained above, and so on, until the method produces no clusters. In this way OSLOM recovers the hierarchical community structure of the original graph.

We will describe next the main features of OSLOM, and what it adds to the state of the art in community detection.

#### Significant clusters

The main characteristic of OSLOM is that it is based on a fitness measure, the score, that is tightly related to the significance of the clusters in the configuration model. In fact, the single cluster analysis is designed to optimize the cluster significance as defined in Ref. [31]. Therefore the output of OSLOM consists of clusters that are unlikely to be found in an equivalent random graph with the same degree sequence. The tolerance 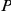, fixed initially, determines whether such clusters are “unexpectedly unlikely”, and therefore significant, or not. So, if the method is fed with a random graph, the output will include very few clusters or even none at all.

#### Homeless vertices

The vertices in a random network will be deemed as homeless. Homeless vertices are those that are not assigned to any cluster. This is a very important feature that OSLOM includes. The presence of random noise or non-significant vertices is an issue that may occur in many real systems. However, very few clustering techniques take into account this possibility. In OSLOM, it comes as a natural output. We will quantitatively analyze this feature when we test the method on benchmark graphs.

#### Overlapping communities

A natural output of OSLOM is the possibility for clusters to overlap. Since each cluster is “cleaned” independently of the others, a fraction of its vertices may belong also to other clusters, eventually. We will show the efficiency of OSLOM in unveiling overlapping vertices in suitably designed benchmarks.

#### Cluster hierarchy

Another relevant feature of OSLOM is the analysis of the hierarchical structure of the clusters. As mentioned above, the third phase of our method includes a procedure to take care of this issue. The results are very good on hierarchical benchmarks.

OSLOM generally finds different depths in different hierarchical branches. In fact, when the algorithm is applied not all vertices are grouped, as some of them are homeless. The coexistence of homeless vertices with proper clusters yields a hierarchical structure with branches of different depths.

#### Weighted networks

OSLOM can be generalized to weighted graphs as well. We assume that the contributions to the probability of having a connection between two vertices 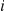 and 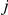 with a certain weight 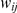, given the vertex degrees 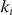 and 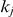 and their strengths, 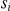 and 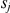, is separable in two different terms in the configuration model: one for the topology and another for the weight [38]. The strength of a vertex is defined as the sum of the weights of all the edges incident on it. We approximate the weight contribution by
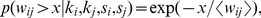
(5)where 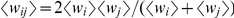 is the harmonic mean of the average weights of vertices 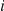 and 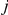, defined as 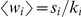 and 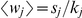, respectively. The idea behind this expression is that the weight of an edge of the null model should be proportional to the average weight of its endvertices. We proposed the harmonic average because it is more sensitive to the small values of 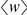.

We use this distribution to define a new variable 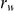, accounting for the probability of having a certain weight on a given edge with the strengths of the vertices and the general weight distribution known. We combine this variable 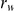 with its topological counterpart, 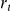, obtaining a new variable 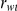. This is a non-trivial task since both probabilities are defined on a different set of elements (see the Supporting Information S1). For 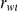 we can estimate, as before, the order statistic distributions and we proceed just as we do for unweighted graphs.

#### Directed graphs

OSLOM can be easily generalized to handle directed graphs. For that, we need to define two uniformly distributed random variables 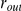 and 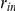. The former is based on the probability that vertex 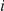 has outgoing edges ending on vertices of the given subgraph 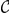, the latter is based on the probability that 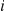 has incoming edges originating from vertices of 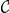. These two probabilities are computed through analogous formulas as in Eq. 1 or numerical approximations to it. The final score of vertex 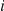 is given by the product 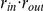. We are able to calculate the distribution of this product and therefore to estimate its order statistics (just as for the weighted case, see Section 1.1. of Supporting Information S1). The rest of the clustering method proceeds as explained above. If graphs have edges with both directions and weights, we have four variables for each vertex: 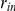, 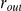 and the corresponding versions for the weights. The final score is given again by the product of these four variables.

#### Dynamical networks

Time-stamped networked datasets are usually divided into snapshots, condensing the relational information between vertices within different time windows. Snapshots are typically analyzed separately, whereas it would be more informative to combine the information from different time slices. For instance, consider two snapshots 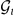 and 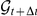 at times 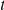 and 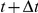, respectively. A simple idea is to find the partition/cover of the network at time 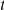, by applying the method to the corresponding snapshot, and to use the result as an input for the application of the method to the network at time 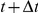. In this way one can see how the community structure at time 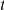 “evolves” to that at time 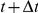. This is a rather general approach, it can be adopted for other algorithms for community detection, like greedy optimization techniques. OSLOM has the useful property that it can start from any initial partition/cover, which can be given as input. In this way the clusters found in 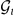 can be used as initial condition for the analysis of 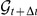. With this approach, the new partition/cover is closer to that in 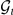 and we are able to track the groups' evolution. Naturally, if the two snapshots are very different from each other (because they refer to times between which the system has changed considerably, for instance), OSLOM produces a partition/cover in 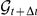 that is uncorrelated with that of 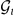.

#### Complexity

**Figure 5:**
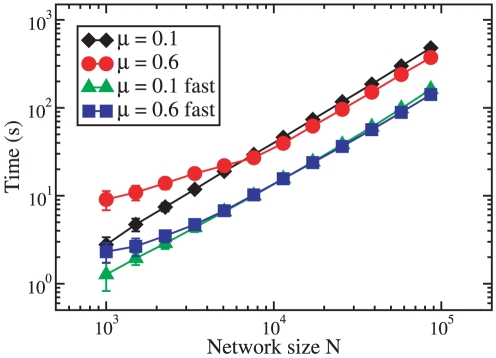
Complexity of OSLOM. The diagram shows how the execution time of two different implementations of the algorithm scales with the network size (expressed by the number of vertices), for LFR benchmark graphs.

**Figure 6:**
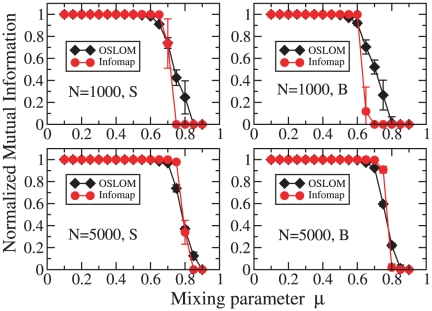
Tests on undirected and unweighted LFR benchmark graphs without overlapping communities. The parameters of the graphs are: average degree 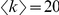, maximum degree 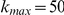, exponents of the power law distributions are 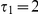 for degree and 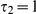 for community size, S and B mean that community sizes are in the range 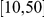 (“small”) and 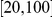 (“big”), respectively. We considered two network sizes: 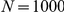 (top) and 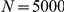 (bottom). The two curves refer to OSLOM (diamonds) and Infomap (circles).

The complexity of OSLOM cannot be estimated exactly, as it depends on the specific features of the community structure at study. Therefore we carried out a numerical study of the complexity, whose results are shown in Fig. 5. We apply the method on the LFR benchmark [41], that we have used extensively to test the performance of OSLOM. We have used both the standard version of the algorithm and a fast implementation, in which the algorithm acts on the partition delivered by a quick method. For each version we have considered undirected and unweighted LFR benchmark graphs with two different levels of mixtures between the clusters (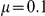 and 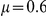, corresponding to well separated and well mixed clusters). The other parameters needed to build the LFR benchmark graphs are the same as for the graphs used in Fig. 6. The diagram of Fig. 5 shows the execution time (in seconds) as a function of the number 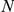 of vertices of the graphs. The processes were run on a workstation HP Z800. The time scales as a power law of 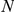 with good approximation, if the graphs are not too small. The behavior seems to depend neither on how mixed communities are, nor on the particular implementation of the algorithm (there seems to be just a factor between the corresponding curves). Power law fits of the large-N portion of the curves yield an exponent 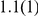, which implies that the complexity is essentially linear in this case.

## Results

### Artificial networks

In this section we test OSLOM against artificial benchmarks, comparing its performance with those of the best algorithms currently available. We mostly adopted the LFR benchmark [41], [42], a class of graphs with planted community structure and heterogeneous distributions of vertex degree and community size. Tests on the well known Girvan-Newman (GN) benchmark [8] are shown in the Supporting Information S1. In this section we present tests on undirected and unweighted networks, with and without hierarchical structure and overlapping communities. We also show how OSLOM handles the presence of randomness in the graph structure. Tests on weighted networks and on directed networks can be found in the Supporting Information S1.

In the following sections, for each network, we compose the results of 10 iterations for the network analysis for the first hierarchical level and the results of 50 iterations for higher levels, if any. The single cluster analysis was repeated 100 times for each cluster.

#### LFR benchmark

The LFR benchmark [41], [42], like the GN benchmark, is a particular case of the *planted* 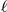 *-partition model* [43], which is the simplest possible model of networks with communities. The planted 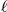-partition model is a class of graphs whose vertices are divided into 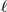 equal-sized groups, such that the probability that two vertices of the same group are linked is 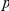, while the probability that two vertices of different groups are linked is 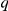, with 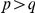. The planted 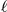-partition model is too simple to describe real networks. Vertices have essentially the same degree and communities have the same size, at odds with empirical analysis showing that both features typically are broadly distributed [19], [44]–[48]. Therefore we have recently proposed a generalization of the model, the LFR benchmark, by introducing power-law distributions for the vertex degree and the community size, with exponents 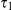 and 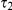, respectively [41]. The LFR benchmark poses a far harder challenge to algorithms than the benchmark by Girvan and Newman, which is regularly used in the literature, and is more suitable to spot their limits. We are of course aware that the communities of the model are still too simple to match the communities of real networks. Other features should be introduced, to tailor the model graphs onto the real graphs. This is certainly doable, and could be specialized to the particular domain of applicability one is interested in. Still, the clusters of the LFR benchmark are a much better proxy of real communities than the clusters of other benchmark graphs.

Vertices of the LFR benchmark have a fixed degree (in this case taken from the given power law distribution), so the two parameters 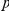 and 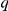 of the planted 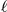-partition model are not independent and we choose as independent variable the *mixing parameter* 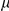, which is the ratio of the number of external neighbors of a vertex by the total degree of the vertex. Small values of 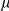 indicate well separated clusters, whereas for higher and higher values communities become more and more mixed to each other.

As a term of comparison we used Infomap [49], which has proved to be very accurate on artificial benchmark graphs [50]. Fig. 6 shows the comparative performance of OSLOM and Infomap on the LFR benchmark, with undirected and unweighted edges and non-overlapping clusters. As a measure of similarity between the planted partition and that recovered by the algorithm we adopted the Normalized Mutual Information (NMI) [51], in the extended version proposed in Ref. [23], which enables one to compare both partitions and covers. We used this definition also for hard planted partitions, since modules found by OSLOM may be overlapping. In all tests on artificial graphs each point is always an average over 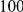 realizations.

The plots correspond to two network sizes, 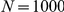 and 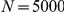, and two ranges of community size, 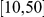 (“small”) and 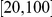 (“big”), that we indicate with the letters S and B, respectively. In this way we can check how much the performance of the algorithm is affected by the network size and the average size of the communities. The other network parameters are given in the caption. From the plots we conclude that OSLOM and Infomap have a basically equivalent performance.

**Figure 7:**
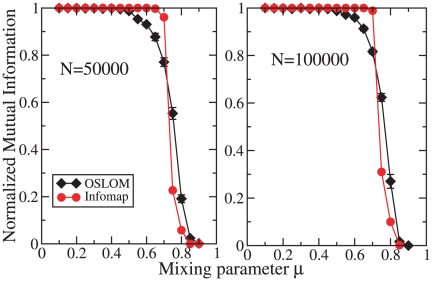
Tests on large undirected and unweighted LFR benchmark graphs without overlapping communities. The network sizes are 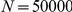 (left) and 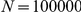 (right), the maximum degree 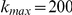 and the community size ranges from 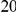 to 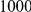. The other parameters are the same as those used for the graphs of Fig. 6. The two curves refer to OSLOM (diamonds) and Infomap (circles).

It is important to test the performance of the algorithms on large graphs as well, given the increasing availability of large networked datasets. The question is if and how their performance is affected by the network size. Fig. 7 shows that both OSLOM and Infomap are effective at finding communities on large LFR graphs. We remark that the inferior accuracy of OSLOM when communities are better defined comes from the fact that the method occasionally finds homeless vertices, i.e. vertices that are not significantly linked to any cluster. These are vertices that happen not to have a significant excess of neighbors within their community with respect to the number of neighbors in the other communities, despite the fact that the average number of internal neighbors is high. This happens because of fluctuations, and the method judges such vertices as not belonging to any group, which makes sense. This issue of the homeless vertices is a general feature of OSLOM. One should not judge it negatively, though. If a vertex 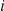 happens to have a number of external neighbors which is appreciably higher than the expected external degree of the vertex 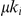, the condition 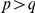 of the planted 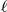-partition model does not hold, so in principle the vertex should not be put in its original community. The confusion derives from the fact that the condition 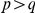 holds *on average*.

#### LFR benchmark with overlapping communities

**Figure 8:**
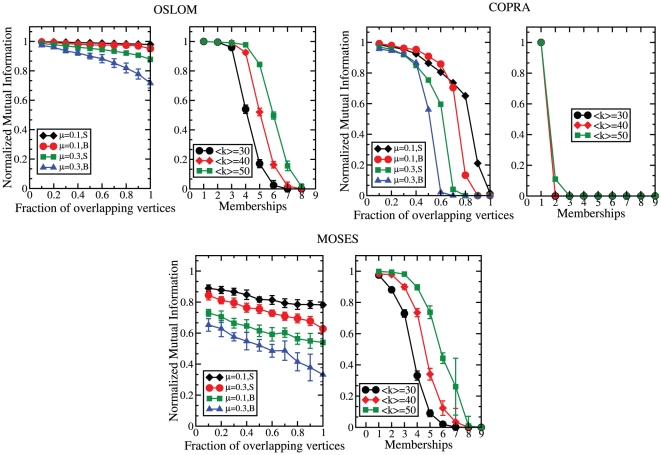
Test on undirected and unweighted LFR benchmark with overlapping communities. The parameters are: 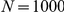, 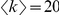, 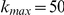, 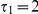, 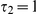. S and B indicate the usual ranges of community sizes we use: 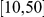 and 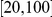, respectively. We tested OSLOM against two recent methods to find covers in graphs: COPRA [52] and MOSES [54]. The left panel displays the normalized mutual information (NMI) between the planted cover and the one recovered by the algorithm, as a function of the fraction of overlapping vertices. Each overlapping vertex is shared between two clusters. The four curves correspond to different values of the mixing parameter 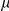 (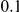 and 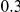) and to the community size ranges S and B. The right panel shows a test on graphs whose vertices are all shared between clusters. Each vertex is member of the same number of clusters. The plot shows the NMI as a function of the number of memberships of the vertices. Each curve corresponds to a given value of the average degree 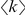. The graph parameters are 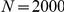, 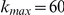, 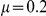, 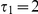, 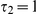. Community sizes are in the range 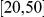.

The LFR benchmark also accounts for overlapping communities, by assigning to each vertex an equal number of neighbors in different clusters [42]. To simplify things, we assume that each vertex belongs to the same number of communities. We cannot use Infomap for the comparison, as it delivers “hard” partitions, without overlaps between clusters. So we used two recent methods, that have a good performance on LFR graphs with overlapping communities: COPRA [52], based on label propagation [53], and MOSES [54], based on stochastic block modeling [55]. COPRA and MOSES are more efficient to detect overlapping communities in LFR benchmark graphs than the popular Clique Percolation Method (CPM) [19], which is the reason why we do not use the CPM here. In Fig. 8 we show how the performance of each method decays with the fraction of overlapping vertices, for different choices of the mixing parameter and for the small (S) and big (B) communities defined above. Since in social networks there may be many vertices belonging to several groups, we also considered the extreme situation of graphs consisting entirely of overlapping vertices. In this case, by increasing the number of memberships of the vertices communities become more fuzzy and it gets harder and harder for any method to correctly identify the modules. From Fig. 8 we deduce that OSLOM significantly outperforms COPRA in both tests and MOSES in the test with overlapping and non-overlapping vertices, while the performances of OSLOM and MOSES are quite close when all vertices are overlapping.

#### Hierarchical LFR benchmark

**Figure 9:**
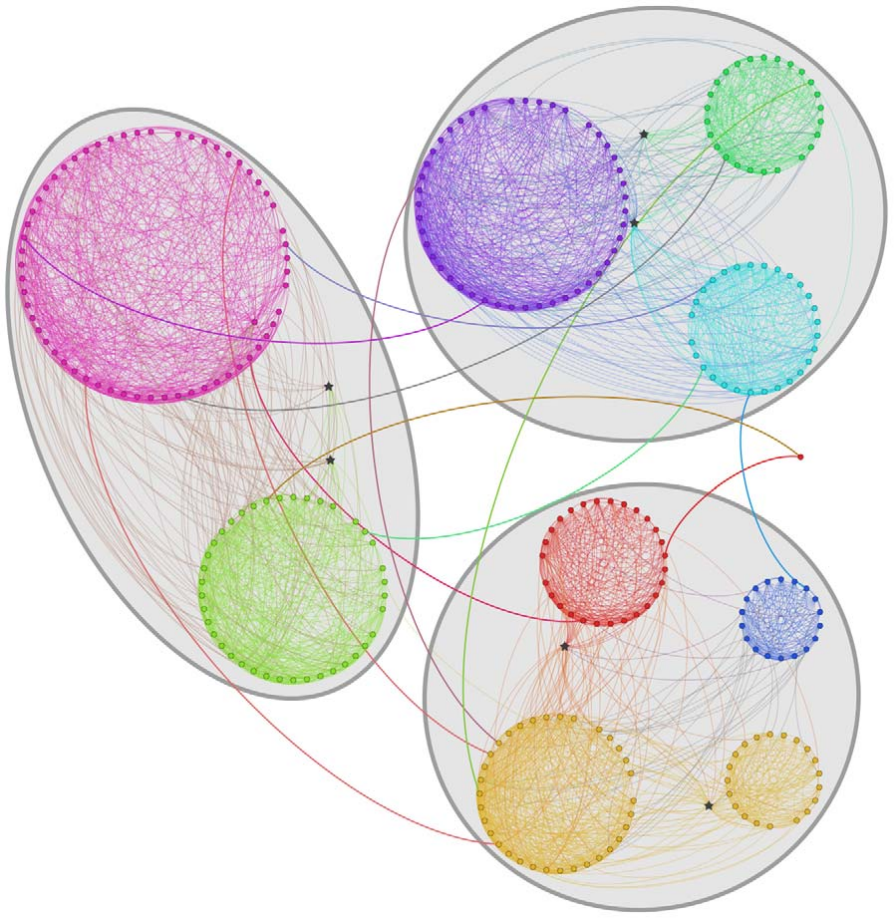
A realization of the hierarchical LFR benchmark with two levels. Stars indicate overlapping vertices.

OSLOM is capable to handle hierarchical community structure as well. To test its performance we have designed an algorithm that produces a version of the LFR benchmark with hierarchy. To keep things simple, we consider a two-level hierarchical structure (Fig. 9). The idea is to use the wiring procedure of the original algorithm twice, first for the micro-communities and then for the macro-communities. In order to do so, we need two mixing parameters: 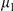, the fraction of neighbors of each vertex belonging to different macro-communities; 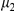, the fraction of neighbors of each vertex belonging to the same macro-community but to different micro-communities.

**Figure 10:**
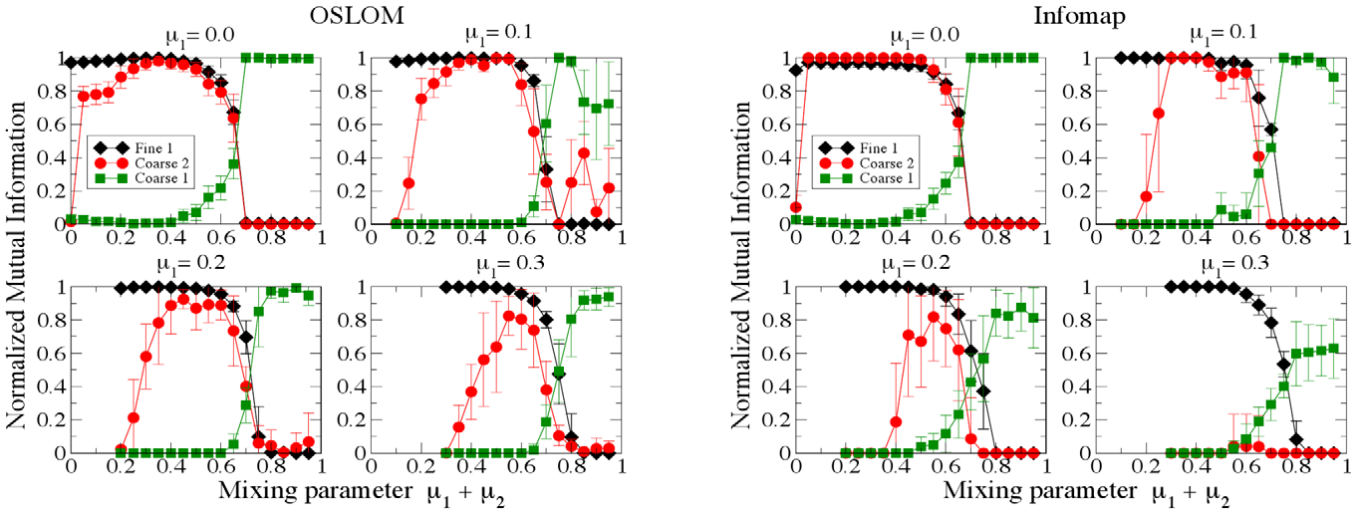
Test on hierarchical LFR benchmark graphs (unweighted, undirected and without overlapping clusters). We compare three pairs of partitions: the lowest hierarchical partition found by the algorithm (indicated by 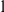) with the set of micro-communities of the benchmark (Fine); the lowest hierarchical partition found by the algorithm with the set of macro-communities of the benchmark (Coarse); the second lowest hierarchical partition found by the algorithm (indicated by 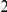) with the set of macro-communities of the benchmark. The corresponding similarities are plotted as a function of 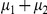, for fixed 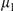. There are 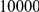 vertices, the average degree 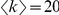, the maximum degree 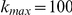, the size of the macro-communities lies between 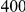 and 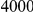 vertices, the size of the micro-communities lies between 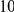 and 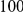 vertices. The exponents of the degree and community size distributions are 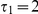 and 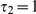.

The question is whether the algorithm is able to recover both planted partitions of the benchmark, which we call *Fine* (micro-communities) and *Coarse* (macro-communities). The partitions found by the algorithm can be one, two or more, we call them partition 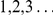. In the test, whose results are illustrated in Fig. 10, we compare the Fine partition with partition 1 (Fine 1), the Coarse partition with partition 2 (Coarse 2), and the Coarse partition with partition 1 (Coarse 1). We compare OSLOM with a recent extension of Infomap to networks with hierarchical community structure [56]. In the plots we show how the similarity of the three pairs of partitions mentioned above varies by increasing 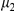 but keeping 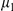 constant (we picked the values 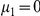, 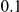, 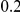, 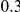). For a better comparison of the panels we put on the x-axis the sum 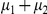, representing the fraction of neighbors of a vertex not belonging to its micro-community. We find that, when 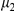 increases, the Fine partition becomes difficult to resolve and, for 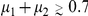, it cannot be found anymore and both algorithms can only find the Coarse partition. Instead, for smaller value of 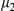, the algorithms can recover both levels. OSLOM performs better than Infomap if 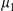 is not too small.

#### Random graphs and noise

We check whether OSLOM is also able to recognize the *absence*, and not simply the presence, of community structure. In random graphs vertices are connected to each other at random, modulo some basic constraints like, e. g., keeping some prescribed degree distribution or sequence. In this way, there are by definition no groups of vertices that preferentially link to each other, so there are no communities. There may be subgraphs with an internal edge density higher than the average edge density of the whole network, but they originate from stochastic fluctuations (noise). A good community finding algorithm should be able to recognize that such subgraphs are false positives, and discard them. Here we want to see if OSLOM distinguishes “order” from “noise”. For this purpose, we carried out two tests.

**Figure 11:**
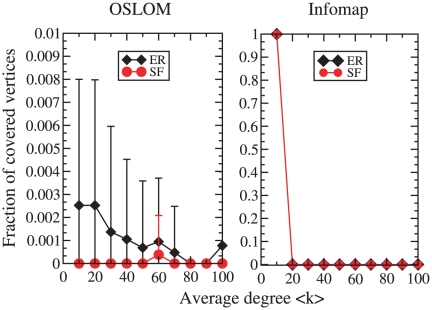
Test on random graphs. We plot the fraction of vertices belonging to non-trivial clusters (i.e. to clusters with more than one and less than 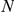 vertices, where 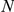 is as usual the size of the graph), as a function of the average degree of the graph. The curves correspond to Erdös-Rényi graphs (diamonds) and scale-free networks (circles). All graphs have 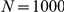 vertices. The only parameter needed to build Erdös-Rényi graphs is the probability that a pair of vertices is connected, which is determined by the average degree 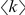. The scale-free networks were built with the configuration model [39], starting from a fixed degree sequence for the vertices obeying the predefinite power law distribution. The parameters of the distribution are: degree exponent 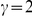, maximum degree 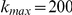.

In Fig. 11 we applied OSLOM and Infomap to Erdös-Rényi random graphs [57] and scale-free networks [58]. The goal is to see whether the algorithms recognize that there are no actual communities. Good answers are the partition with as many communities as vertices, or the partition with all vertices in the same community. Let us call 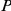 the partition found by the algorithm at hand. Clusters in 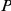 containing at least two vertices and smaller than the whole network indicate that the method has been fooled. The fraction of graph vertices belonging to those clusters is a measure of reliability: the lower this number, the better the algorithm. In Fig. 11 we show this variable as a function of the average degree 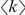 of the random graphs we considered. For OSLOM it remains very low for all values of 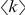. This is not surprising, since OSLOM estimates the statistical significance of clusters, and is therefore ideal to detect stochastic fluctuations. Infomap instead finds many non-trivial clusters when 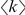 is low, whereas it correctly recognizes the absence of community structure if 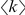 increases.

**Figure 12:**
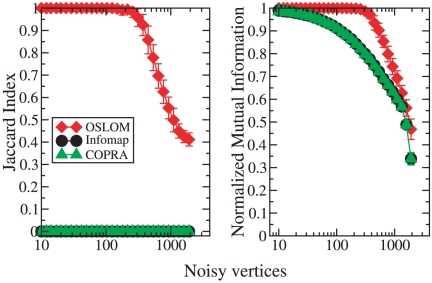
Test on graphs including communities and noise. The communities are those of an LFR benchmark graph (undirected, unweighted and without overlapping clusters), with 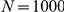, 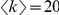, 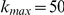, 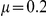. The cluster size ranges from 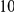 to 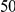 vertices. The noise comes by adding vertices which are randomly linked to the existing vertices, via preferential attachment. The test consists in checking whether the community finding algorithm at study (here OSLOM, Infomap and COPRA) is able to find the communities of the planted partition of the LFR benchmark and to recognize as homeless the other vertices.

The second test deals with graphs consisting of an *ordered* part, with well-defined clusters, and a *noisy* part, consisting of vertices randomly attached to the rest of the network. The ordered part is an LFR benchmark graph with 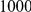 vertices and represents the starting configuration of our system. The noisy vertices (up to 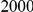 in number) are successively added in sequence, and a newly added vertex is linked to the other ones via preferential attachment [58]. The initial degree of the noisy vertices is drawn from a power law distribution with 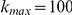 and exponent 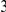. We measure two things, as a function of the number of noisy vertices: the similarity between the set of noisy vertices and the set of homeless vertices found by OSLOM, which is expressed by the Jaccard Index [59] (Fig. 12, left); the similarity between the planted partition of the ordered part of the graph and the subset of the partition found by OSLOM including (only) the vertices of the ordered part, which is expressed by the normalized mutual information (Fig. 12, right). We compare OSLOM with Infomap and COPRA [52]. We find that OSLOM correctly separates the clusters and the noise up to a number of about 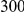 noisy vertices, which represent almost a third of the whole network. Infomap and COPRA, instead, do not recognize the noisy vertices, no matter how small their number is. Also, they tend to mix noisy vertices with the clusters of the planted partition of the ordered part, as shown by the fact that the partition they recover never exactly match the planted partition, not even when just a few noisy vertices are present. These results are actually understandable in the case of Infomap, which is based on the minimization of the code length required to describe random walks taking place on the graph: singletons (clusters consisting of single vertices) are generally not admitted because they increase the amount of information required to map the process, due to the high number of transitions of the walker from the singletons to the rest of the graph and back.

### Real networks

In this section we discuss the application of OSLOM to networks from the real world. In Table 1 we list the networks considered in our analysis, along with some basic statistics obtained from the detection of their community structure with OSLOM.

**Table 1:** Basic statistics of the real networks we analyzed, including the main features of their community structure, detected by OSLOM.

Network	N	E	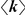	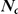	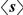	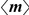	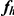
Zachary's club	34	78	4.59	2	17.0	1.03	0.0294
Dolphins	62	159	5.13	2	32.5	1.08	0.0322
Football	115	613	10.7	11	10.0	1.00	0.0434
UK commuting	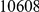	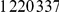	230.07	248	45.43	1.06	0.00386
C. elegans	453	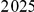	8.94	25	17.04	1.22	0.229
Word association	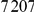	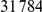	8.82	261	22.48	1.35	0.395
Live Journal	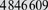	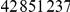	17.6	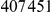	10.01	1.19	0.294
www.uk	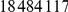	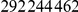	15.81	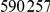	28.08	1.02	0.125
US airports 2009 (jan)	448	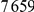	34.19	11	33.81	1.28	0.352
US airports 2009 (mar)	456	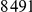	37.24	6	67.83	1.22	0.272
US airports 2009 (jun)	453	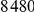	37.42	9	45.33	1.28	0.315
US airports 2009 (sep)	452	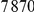	34.81	9	41.55	1.26	0.347

From left to right, we list the number of vertices 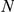 and edges 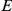, the average degree 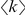, the number of clusters 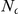, the average cluster size 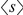, the average number of memberships per vertex 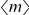 and the fraction 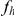 of vertices not assigned to any cluster (homeless vertices). The values related to the community structure refer to the lowest hierarchical level.

We analyzed different types of systems: social, information, biological and infrastructural networks. Here we discuss only some of them, the rest of the analysis can be found in the Supporting Information S1.

#### The word association network

**Figure 13:**
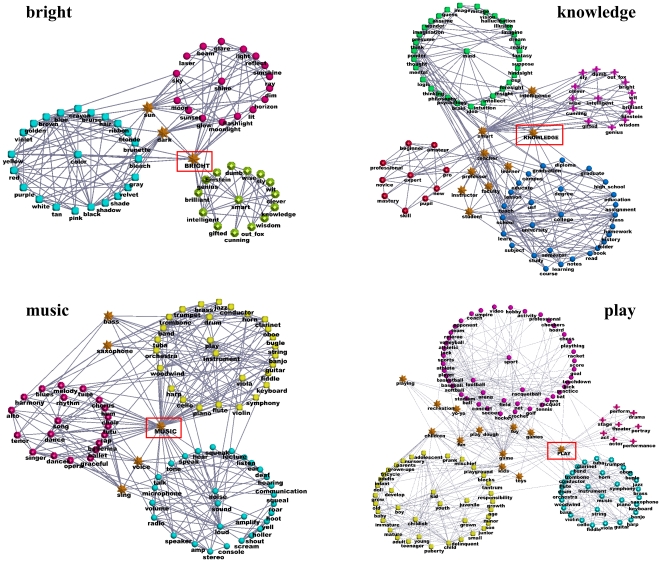
Application of OSLOM to real networks: the word association network. Stars indicate overlapping vertices.

This network is built on the University of South Florida Free Association Norms [60]. Here the presence of an edge between words 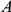 and 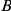 indicates that some people associate 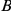 to the word 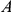. This network is considered a paradigmatic example of graph with overlapping communities [19], since several words may have various meanings and belong to different groups of words. In Fig. 13 we see a few subgraphs of the word association network, revolving around four keywords: *bright*, *knowledge*, *music* and *play*. We see that the keywords are shared among several clusters, which are semantically highly homogeneous. For instance, *bright* belongs to three groups, centered on the words *color*, *shine* and *smart*, respectively, which makes sense. In the same subgraph, the words *sun* and *dark* are also overlapping vertices, belonging to the groups of *color* and *shine*, as one might expect. In the subgraph centered on *knowledge*, one distinguishes the groups referring to the words *mind*, *intelligent*, *expert* and *college/university*. Here there are many overlapping vertices, like the word *intelligence*, shared between the groups of *mind* and *intelligent*, and a bunch of terms indicating (mostly) professional status within schools and/or universities, like *student*, *professor*, *teacher*, etc., which lie between the groups of *expert* and *college/university*. In the third subgraph, the word *music* is shared by the groups of *instrument*, *song/dance* and *noise/sound*: other overlapping vertices are the words *sing* and *voice*, lying between *song/dance* and *noise/sound*, and the words *bass* and *saxophone*, belonging to the groups of *song/dance* and *instrument*. Finally, the word *play* sits between the communities of *sport*, *music* and *youth/kid*; other overlapping vertices in this subgraph include *game*, *children*, *toy*, etc.

#### UK commuting

**Figure 14:**
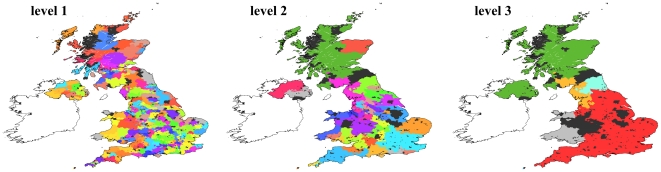
Application of OSLOM to real networks: flows of commuters in the UK. Black points indicate overlapping vertices.

This is the network of flows of commuters between areas of the United Kingdom, and therefore it has a clearly geographic character. It is composed of 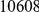 vertices, each representing a ward, i. e. a geographical division used in the UK census for statistical purposes. The whole territory of the United Kingdom is divided into wards. Each edge corresponds to a flow of commuters between the ward of origin and that of destination, with a weight accounting for the number of commuters per day. The data were collected during the 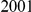 UK census, when the ward of residence and the ward of work/study was registered for a sizeable part of the British population. The database can be accessed online at the site of the Office for National Statistics http://www.ons.gov.uk/census. OSLOM finds three hierarchical levels (Fig. 14). The clusters of the second level delimit geographical areas typically centered about one major town. In the highest level the areas of England, Wales, Scotland and Northern Ireland are clearly recognizable. Interestingly, Northern Ireland and Scotland are parts of the same community, due to the large flow of commuters between the two regions, despite the geographical separation. Black points represent overlapping vertices.

#### LiveJournal and UK Web

We also applied OSLOM to two large networks. The first is a network of friendship relationships between users of the on-line community *LiveJournal* (www.livejournal.com), and was downloaded from the Stanford Large Network Dataset Collection (http://snap.stanford.edu/data/). The second is a crawl of the Web graph carried out by the Stanford WebBase Project (http://dbpubs.stanford.edu:8091/~testbed/doc2/WebBase/), within the UK domain (.uk). We remind that the Web graph is a directed graph whose vertices are Web pages, while the edges are the hyperlinks that enable one to surf from one page to another. These two systems are too large for OSLOM, due to the huge variety of possible cluster sizes to explore. Therefore we applied a two-step method: in the first step, we derived an initial partition 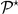 with the Louvain method [61], which is able to handle large networked datasets; in the second step, we apply OSLOM to refine the clusters of 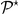. In principle, this procedure should yield the same partitions/covers as applying OSLOM directly, if one repeated OSLOM's cluster search many times. But this would make the calculations too lengthy, so, in order to complete the analysis within a reasonable time, it is necessary to keep the number of iterations low. In this way there is the big advantage of drastically reducing the computational complexity, which makes large systems tractable, even if results would be more accurate if one could apply OSLOM from scratch. Clearly, since different iterations are independent processes, one could sensibly increase the statistics by distributing the iterations among different processors, if available.

**Figure 15:**
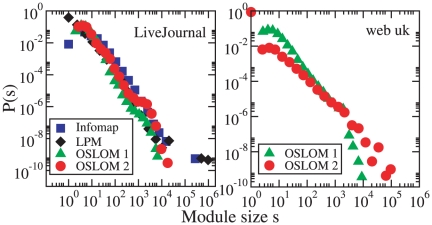
Application of OSLOM to real networks: friendships of *LiveJournal* users (left) and sample of the .uk domain of the Web graph (right). We show the distribution of cluster sizes obtained by OSLOM for the first two hierarchical levels (OSLOM 1 and OSLOM 2). For *LiveJournal* we can compare the distributions with those found with Infomap [49] and the Label Propagation Method (LPM) by Leung et al. [62].

In Fig. 15 we present the distribution of cluster sizes of the first two hierarchical levels found by OSLOM. The results are obtained by performing a single iteration on a workstation HP Z800. For the Web graph, which is the larger system, with nearly 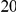 million vertices and 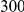 million edges (see Table 1), the analysis was completed in about 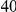 hours. For the social network of *LiveJournal* we can compare the results with the corresponding distributions found by Infomap and the Label Propagation Method (LPM) proposed by Leung et al. [62], which were computed in a recent analysis [48]. In that work the original Infomap was used, so neither Infomap nor the LPM could detect hierarchical community structure and there is just one cluster size distribution, corresponding to the single partition recovered. The distributions are broad and quite similar across different methods. Interestingly, the two hierarchical levels of *LiveJournal* (OSLOM 1 and OSLOM 2) are not too different, indicating a sort of self-similarity of the community structure. For the Web the two levels are more dissimilar and the distributions have a clear power law decay (with different exponents) up to a cutoff, which is approximately the same for both curves (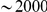 vertices).

#### Dynamic datasets: the US air transportation network

**Figure 16:**
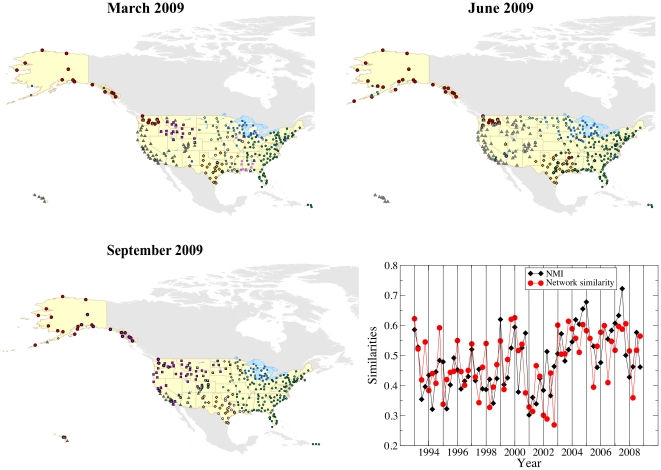
Application of OSLOM to real networks: US airport network. The maps show the position of the airports, which are represented by symbols, indicating the communities found by applying OSLOM directly to the corresponding network, without exploiting the information of previous snapshots. The diagram shows the “seasonality” of air traffic. The normalized mutual information (diamonds) was computed comparing the cover of the system at time 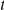 adjusted by OSLOM on the network at time 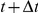, and the cover obtained by applying OSLOM directly to the system at time 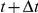. The circles are estimates of the similarity of the network matrices of snapshots separated by 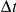 (one year). For each year we took four snapshots, by cumulating the traffic of each trimester. The most stable networks are typically in winter (vertical lines).

For the last application, we used a time-stamped dataset, the US air transportation network. The data can be downloaded from the Bureau of Transportation Statistics (US government) (http://www.bts.gov). Vertices are airports in the USA and edges are weighted by the number of passengers transported along the corresponding routes. In Fig. 16 we show the geographical location of the airports and their communities, indicated by the symbols, for three snapshots, corresponding to the traffic in March, June and September 2009, respectively. We remind that for dynamical datasets we usually take the partition/cover 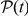 of the system at time 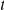, and we use it as initial partition/cover for the topology of the system at time 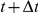, which is then refined by OSLOM, in order to “adapt” 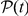 to the current structure. This is done to exploit the information of more snapshots at the same time. Since the three maps of Fig. 16 are mostly illustrative, communities were derived by applying directly OSLOM to the corresponding snapshots, for simplicity. The diagram indicates the similarity between networks and their corresponding partitions/covers in different snapshots. Each snapshot represents the whole traffic of one trimester, which corresponds to a season, while 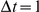 year, as we want to measure the variation of the network structure in consecutive seasons. The similarity between partitions/covers is computed with the normalized mutual information, as usual. The similarity of two weighted networks like the ones at study is measured in the following way. First, one computes the distance 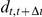 between the matrices 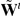 and 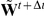: 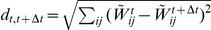. The matrix 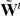 is derived from the standard weight matrix 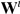 by dividing each edge weight by the sum of all edge weights. This is done because the traffic flows tend to increase steadily in time, so comparing the original weight matrices is not appropriate. The quantity 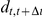 is a dissimilarity measure. We turn it to a similarity index by changing its sign, adding a constant and rescaling the resulting values. Since we wish to compare the trend of the network similarity with that of the partition/cover similarity, the additional constant and the rescaling factor are chosen such to reproduce the average and the variance of the curve of the normalized mutual information. After this operation, the two trends are finally comparable. The diagram shows that both measures follow a yearly periodicity, with peaks corresponding to the winter season, which is then more stable than the others.

## Discussion

We have introduced OSLOM, the first method that finds clusters in networks based on their statistical significance. It is a multi-purpose technique, capable to handle various types of graphs, accounting for edge direction, edge weights, overlapping communities, hierarchy and network dynamics. Therefore, it can be used for a wide variety of datasets and applications.

We have thoroughly tested OSLOM against the best algorithms currently available on various types of artificial benchmark graphs, with excellent results. In particular, OSLOM is superior on directed graphs and in the detection of strongly overlapping clusters. Moreover, it is an ideal method to recognize the absence of community structure and/or the presence of randomness in graphs. In some cases OSLOM returns slightly less accurate results than other methods, because it finds several homeless vertices when communities are fuzzy. This is due to the fact that, in the realizations of benchmark graphs, it may happen that some vertices end up having the same number of neighbors (or even more) in other communities than in their own, due to fluctuations, even if on average this does not happen. So, the classification of those vertices, *imposed* by the planted 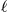-partition model, is not justified topologically. This is an important general issue that needs to be assessed in the future, to avoid systematic errors in the testing procedure.

OSLOM is a local algorithm, so it respects the nature of community structure, which is a local feature of networks, the more so the larger the systems at study. However, the null model adopted to estimate the statistical significance of clusters is the configuration model, which is global. This is the same null model adopted in modularity optimization [63], and is responsible for the serious problems of this technique, like its well known resolution limit [64]. Therefore we perform an iterative cluster search within the clusters found after the first application of the method, by considering each cluster as a network on its own. In this way we progressively limit the horizon of the part of the network under exploration, and we are able to find the smallest significant clusters, which are the natural building blocks of the network and the basis of its hierarchical community structure. So the null model, originally global, gets confined to smaller and smaller portions of the graph. The actual resolution of the method is thus not due to the null model, but to the choice of the threshold 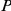. In this paper we have set 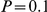, which is often used in various contexts and delivers an excellent performance on the benchmark graphs we have adopted. Nevertheless, how much a real graph *deviates* from a random graph depends on the specific system at hand, and it would be more appropriate to estimate the threshold 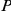 case by case. This is an issue to consider for future work. We remark that also for modularity optimization one could in principle iteratively restrict the null model to the clusters found by the method. However, modularity is based on the *expected* value of variables estimated on the null model, neglecting random fluctuations, which is why modularity can attain large values on specific partitions of random graphs [65]–[67]. OSLOM instead accounts for those fluctuations, so it is far more reliable, in this respect. Furthermore OSLOM is a local method, so it does not suffer from the severe problems coming from modularity's global optimization [68].

Another important aspect to emphasize is the need to perform many iterations, to get more accurate results. This is not a specific feature of OSLOM, but it should be done for all community detection techniques with a stochastic character, like methods based on optimization (e. g., modularity optimization). In the literature there is the general attitude to perform a single iteration, and to reduce the complexity of an algorithm to the time required to carry out one iteration. But this is not appropriate, especially on large networks. For instance, by performing a single iteration, vertices lying on the border between clusters may be assigned to a specific cluster, while in many cases they are overlapping. By combining the results of several iterations, instead, it is more likely to distinguish overlapping vertices from the others. Furthermore, one can compute the strength of the membership of vertices in different clusters, from the frequency with which they were classified in each cluster. One can also disambiguate stable from unstable clusters, which could be recovered from specific iterations. So, it is crucial to collect and combine the results of many iterations. Of course, the complexity of the method grows with the number of iterations, but it can be considerably reduced by distributing runs among many different processors, if large computer clusters are available.

The running time of OSLOM is dominated by the exhaustive search of significant vertices, inside and outside the clusters. This search could be carried out with greedy approaches, with a huge computational advantage, and this is an improvement we plan to implement in the near future. On the other hand, if one wishes to attack very large graphs, OSLOM could be used at a second stage, as a refinement technique, to clean the results of an initial partition delivered by a fast algorithm. In this case, since the initial clusters are usually cores or parts of the significant clusters we are looking for, OSLOM converges far more rapidly than its direct application without inputs. We have seen in the previous section that, by combining OSLOM with the Louvain method by Blondel et al., we were able to handle systems with millions of vertices.

We have proposed a recipe to deal with the increasingly more important issue of detecting communities in dynamic networks. The idea is to take advantage of the information of different snapshots at the same time, by “adapting” the partition/cover of the earlier snapshot to the topology of the other one. In this way it is possible to uncover the correlation between the structures of the system at different time stamps.

We have shown the versatility of OSLOM by applying it to various networked datasets. OSLOM provides the first comprehensive toolbox for the analysis of community structure in graphs and is an ideal complement of existing tools for network analysis. The algorithm, with all its variants (including a fast two-step procedure for the analysis of very large networks) is implemented in a freely downloadable and documented software (http://www.oslom.org).

## Supporting Information

Supporting Information S1 (PDF)
