# Supplementary material for: Finding Statistically Significant Communities in Networks
Source: PLoS One. 2011 Apr 29;6(4):e18961. doi: 10.1371/journal.pone.0018961 (PMC3084717; doi:10.1371/journal.pone.0018961)
Supplement: Supporting Information S1 — (PDF) [file pone.0018961.s001.pdf]

Supporting Information S1
Finding statistically significant communities in networks

A. Lancichinetti, F. Radicchi, J.J. Ramasco & S. Fortunato

1 Numerical estimation of the internal connection probability

The assessment of a cluster's significance given the null (configuration) model relies on the estimation of the probability described in Eq. 1 of the main text. This function has to be evaluated many times along the execution of OSLOM in order to clean up each cluster and to evaluate the clusters at the different hierarchical levels. We explain here how the values of the distribution function can be estimated or approximated in a practical implementation of OSLOM.

For convenience, we rewrite the equation here

$$p(k_i^{in}|i, \mathcal{C}, \mathcal{G}) = A \frac{2^{-k_i^{in}}}{k_i^{out}!\; k_i^{in}!\; (m_{\mathcal{C}}^{out} - k_i^{in})!\; (M^*/2)!}. \tag{S1}$$

While estimating the value of the probability of Eq. S1 for a certain k_i^{in}, the most computationally expensive part is the evaluation of the normalization factor A. In fact, this would force us to evaluate the rest of the formula for all the allowed values of k_i^{in} and add up the result. A simple way out of this problem is to approximate the distribution by another whose normalization factor is known. To do so, we can think of a slightly different null model, in which the edges are still drawn at random and the formation of self-loops is admitted. This is actually the null model on which the definition of modularity is based [1]. In such model, the equivalent of Eq. S1 becomes an hypergeometric function that is much easier to estimate (see [2]). Both distributions, that of Eq. S1 and the hypergeometric, provide close numerical values for the same k_i^{in}, except if the probability of generating self-loops in the null model is high. The probability that reshuffling the connections at random a stub of vertex i connects to another stub of the same vertex, is given by $k_i^2/2M$. In the software implementation of OSLOM, the hypergeometric approximation for Eq. S1 is used as long as $k_i^2/2M < 1$. Otherwise, we directly measure A from Eq. S1.

2 Extension of the method to weighted networks

In the main text, it is briefly discussed how to extend OSLOM to weighted graphs. We mention also that some of the technical issues, such as combining both r_w and r_t, are not trivial. This procedure is described here in further detail.

Remember that we start from an ansatz for the distribution of the weights in the null model. The distribution of the probability of having a certain weight on the edge joining vertices i and j was assumed to be

$$p(w_{ij} > x|k_i, k_j, s_i, s_j) = \exp(-x/\langle w_{ij}\rangle). \tag{S2}$$

The idea behind this expression is that the weight of an edge is proportional to the average weight of its endvertices ($\langle w_i =\rangle s_i/k_i$ and $\langle w_j\rangle = s_j/k_j$). We proposed the harmonic average because it is more sensitive to small values of $\langle w_i\rangle$. Our goal is to define a fitness function r which has to be a uniform

random variable on our randomized weighted network. And we want to combine the fitness function depending on the topology with one depending on the weight distribution in order to detect meaningful fluctuations in any of them.

Let us consider a vertex i which has l connections with a given subgraph $\mathcal{C}$ (not including i). For the topological part, we have already computed the probability that i shares l or more edges with vertices of $\mathcal{C}$ (Eq. S1). We call this number r_t. Each of the l edges joining i with $\mathcal{C}$ carries a weight. We consider the corresponding normalized weight $\omega_s = w_s/\langle w_s \rangle$, where w_s is the weight on the s-th edge, with $s = 1, 2, \ldots, l$. Since we want a single number taking into account all the weights in the set, we can simply consider the sum of all the ω_s:

$$\Omega = \sum_{s=1}^{l} \omega_s \tag{S3}$$

Ω is the sum of l exponentially distributed variables (with rate equal to one) and therefore it follows the Erlang distribution [3]. Let us call r_w the cumulative of Ω:

$$r_w = p(\Omega > x) = e^{-x} \sum_{q=0}^{l-1} x^q/q! \tag{S4}$$

In this way, we managed to define two variables r_t and r_w which are both uniformly distributed in the null model. Now, we would like to combine these two scores to have a final score for our vertex i. Unfortunately this is not so simple. We remind that r_w is defined only on the N_n neighbors of subgraph $\mathcal{C}$ while r_t is defined for all the $N^* = N - n_{\mathcal{C}} \geq N_n$ vertices out of $\mathcal{C}$, so the two variables are defined on samples of different size, in general. A way to overcome this difficulty is to scale r_t to an equivalent random variable ${r'}_t$ defined on a smaller sample. This amounts to map each index i in the set $1, 2, ..., N^*$ of the old variable onto an index j in the set $1, 2, ..., N_n$ of the new variable. Given i, the natural solution is to pick the index j such that the cumulative probability Ω_q^t on the sample of N^* vertices coincides (at least with the approximation allowed by the specific numerics involved) with the cumulative probability Ω_q^w on the smaller sample of N_n vertices. It can be shown that this can be achieved with a good approximation (in the limit of j close to N_n) with the following rescaling:

$${r'}_t = r_t \cdot \frac{N^* + 1}{N_n + 1}. \tag{S5}$$

Once we computed ${r'}_t$ and r_w we need to combine them in order to have a single score to rank the vertices. We consider the product ${r'}_t \cdot r_w$ and the final score $r_{tw} = p({r'}_t \cdot r_w < x) = x(1 - \log x)$. The last expression comes from the assumption that the two variables are both uniform and independent. The set of variables $\{r_{tw}\}$ is then used to rank the vertices and to compute the cumulative probabilities Ω_q^{tw}, with N_n instead of N^*.

3 Further tests on benchmark graphs

3.1 Girvan-Newman benchmark

The benchmark by Girvan and Newman [4] (GN benchmark) is a class of graphs with 128 vertices, each, divided into four equal-sized groups. Every vertex has expected degree 16 (with a very peaked distribution about 16). The (average) number of neighbors of a vertex within its group is k_{in}, whereas the (average) number of external neighbors is k_{out}. By construction, $k_{in} + k_{out} = 16$. In the language of the planted ℓ-partition model [5], the probability that a vertex is linked to another vertex of its group is $p = k_{in}/31$, the probability that a vertex is linked to external vertices is $q = k_{out}/96$. The condition

$p > q$ for the four groups to be communities is then equivalent to $k_{out} \lesssim 12$ (this does not account for random fluctuations, though [2, 6]).

Fig. S1 shows the Normalized Mutual Information (in the version devised in Ref. [7]) between the planted partition of the GN benchmark and the partition found by the algorithm as a function of k_{out}. As a term of comparison we used again Infomap [8]. Fig. S1 shows that Infomap is more accurate for low values of k_{out} than OSLOM, but its performance drops rapidly for $k_{out} \gtrsim 6$, whereas OSLOM shows a slower decay.

OSLOM is slightly worse than Infomap because it finds several homeless vertices, as we explained in the main text (Section 3.1.1).

3.2 Weighted LFR benchmark

In Figs. S2 and S3 we report the comparative analysis of OSLOM and Infomap on weighted LFR graphs. To build the weighted benchmark graphs [9] one needs two additional parameters: the exponent β of the relation between the strength of a vertex and its degree (the strength of a vertex is the sum of the weights of the edges incident on the vertex); the weighted mixing parameter μ_w, which is the natural extension to weighted networks of the topological μ (that here we call μ_t), i.e. it is the ratio between the sum of the weights on the edges joining a vertex to its neighbors in different communities and the strength of the vertex. In the analysis, we fix the value of the topological mixing parameter μ_t and see how the normalized mutual information varies as a function of μ_w. In Fig. S2 the benchmark graphs consist of 5000 vertices, and we consider the usual two ranges of community sizes (S and B). In Fig. S3 the graphs consist of 50000 vertices, and we consider a single, but much wider, range of community sizes (from 20 to 1000). When $\mu_t = 0.5$ or $\mu_t = 0.6$, we find that OSLOM detects the right clusters for any value of μ_w, for $N = 5000$, which is truly remarkable, while Infomap is unable to find the partition for $\mu_w \gtrsim 0.6$. OSLOM's striking result comes from the fact that the score r_{tw} of a vertex on weighted graphs is given by the product of two numbers, the topological score r'_t and the weight score r_w (Section 2). If μ_t is not too large, the topological term r'_t is very low and brings down the whole score r_{tw}, which remains significant for any choice of the weighted mixing parameter μ_w. Basically, OSLOM is able to recognize the right clusters from the topology alone. When $\mu_t = 0.5$ or $\mu_t = 0.6$ and $N = 50000$, OSLOM maintains an excellent performance for the whole range of μ_w, while Infomap again fails for $\mu_w \gtrsim 0.6$. For $\mu_t = 0.7$ the performances of the two algorithms worsen and OSLOM is still superior, though the results are essentially comparable for both network sizes. For $\mu_t = 0.8$ Infomap is more accurate than OSLOM, when $N = 5000$, while both methods are not very good when $N = 50000$. However, from Figs. S2 and S3 it is apparent that OSLOM works the better, the larger the network size. So, on very large networks ($N \gg 50000$) we expect that OSLOM has a comparable or superior performance than Infomap for every pair of values (μ_t, μ_w). We also infer that the performance of both algorithms worsens if clusters are on average larger.

3.3 Directed LFR benchmark

Figs. S4 and S5 show the results of the test on directed LFR graphs [9]. This time we have to distinguish between in-degree (number of incoming edges) and out-degree (number of outgoing edges) of a vertex. The in-degree distribution is taken to be a power law, with exponent τ_{in}, whereas the out-degree is the same for all vertices, for simplicity. The mixing parameter μ expresses the ratio of the number of in-neighbors of a vertex belonging to different clusters and the total number of in-neighbors of the vertex. The in-neighbor of a vertex i is any vertex j connected to i by an edge going from j to i. Figs. S4 and S5 tell us that OSLOM outperforms Infomap, especially when communities span a broader range of sizes. The performances of both algorithms slightly worsen on larger networks.

4 Real-world systems

4.1 Zachary karate club

The famous karate club network of Zachary [10] is a standard benchmark in community detection. Vertices are members of a karate club in the United States, who were monitored during a period of three years. Edges connect members who had social interactions outside the club. After some time, a conflict between the club president and the instructor caused the fission of the club in two separate groups, supporting the instructor and the president, respectively. In Fig. S6 we see the community structure found by OSLOM. It indeed finds two communities, plus a homeless vertex (12). Vertex 3 is shared between the two clusters, as it has several neighbors in both groups. We shall illustrate overlapping and homeless vertices with stars and triangles, respectively. The communities coincide with the ones observed by Zachary with the exception of vertices 3 and 12, which Zachary put with the squares. However, vertex 3 is overlapping, so it belongs to both clusters, which seems quite reasonable by looking at the figure. Also, vertex 12 is homeless due to its loose relationship with its group (it has only one neighbor).

4.2 Dolphin social network

Fig. S7 presents OSLOM's results for the network of bottlenose dolphins living in Doubtful Sound (New Zealand). The network was compiled by Lusseau [11]. Vertices of the network are dolphins and two dolphins are connected if they were seen together more often than expected by chance. The dolphins separated in two groups after one of them left the place for some time. OSLOM finds two communities, with five overlapping vertices (2, 8, 20, 29, 31), plus two homeless vertices (40, 61), which are very loosely connected to the rest of the graph. All vertices which are uniquely assigned to the same group (indicated by the same symbol, square or circle, in the figure) are classified in the same community by Lusseau as well.

4.3 American college football

Another well known benchmark in community detection is the network of American college football teams, compiled by Girvan and Newman [4]. It comprises 115 vertices, representing Division I-A colleges. Edges correspond to games played by the teams against each other during the regular season of fall 2000. The teams are divided into 12 conferences. Games between teams in the same conference are usually (but not always) more frequent than games between teams of different conferences, so there is a organization in clusters where communities correspond to conferences. In Fig. S8 we see that OSLOM finds three hierarchical levels. The lowest level consists of 11 clusters and 5 homeless vertices. There are no overlapping vertices. Six clusters correspond exactly to the conferences, three others match the conferences up to one vertex, one up to two vertices, the last cluster along with the homeless vertices mostly mix teams of the conferences Sun Belt and Independents. The latter is not a proper conference, whereas Sun Belt includes colleges which are geographically very spreadout, so they happen to play quite often games with the other teams, resulting much more mixed with them than teams of other conferences. Interestingly, in the second hierarchical level we find two large communities (plus four homeless teams), corresponding quite well to a geographical separation of the colleges in East and West.

4.4 *C. elegans* metabolic network

Fig. S9 presents the community structure of the metabolic network of *C. elegans*. The network has been compiled by Duch and Arenas [12] and it has been often used in applications of community detection algorithms. Here vertices are metabolites and edges connect pairs of metabolites involved in at least one biochemical reaction. OSLOM finds two hierarchical levels, the lower with 25 clusters, the higher with

3 (but one of them is much smaller than the other two). The fraction of homeless vertices in the lower level is larger than 20% (see Table 1 of main text) and the network appears therefore rather “noisy”.

References

1. Newman MEJ, Girvan M (2004) Finding and evaluating community structure in networks. Phys Rev E 69: 026113.

2. Lancichinetti A, Radicchi F, Ramasco JJ (2010) Statistical significance of communities in networks. Phys Rev E 81: 046110.

3. Evans M, Hastings N, Peacock B (2000) Statistical Distributions. New York: Wiley-Interscience.

4. Girvan M, Newman ME (2002) Community structure in social and biological networks. Proc Natl Acad Sci USA 99: 7821–7826.

5. Condon A, Karp RM (2001) Algorithms for graph partitioning on the planted partition model. Random Struct Algor 18: 116–140.

6. Bianconi G, Pin P, Marsili M (2009) Assessing the relevance of node features for network structure. Proc Natl Acad Sci USA 106: 11433–11438.

7. Lancichinetti A, Fortunato S, Kertész J (2009) Detecting the overlapping and hierarchical community structure in complex networks. New J Phys 11: 033015.

8. Rosvall M, Bergstrom CT (2008) Maps of random walks on complex networks reveal community structure. Proc Natl Acad Sci USA 105: 1118-1123.

9. Lancichinetti A, Fortunato S (2009) Benchmarks for testing community detection algorithms on directed and weighted graphs with overlapping communities. Phys Rev E 80: 016118.

10. Zachary WW (1977) An information flow model for conflict and fission in small groups. J Anthropol Res 33: 452-473.

11. Lusseau D (2003) The emergent properties of a dolphin social network. Proc Royal Soc London B 270: S186-S188.

12. Duch J, Arenas A (2005) Community detection in complex networks using extremal optimization. Phys Rev E 72: 027104.

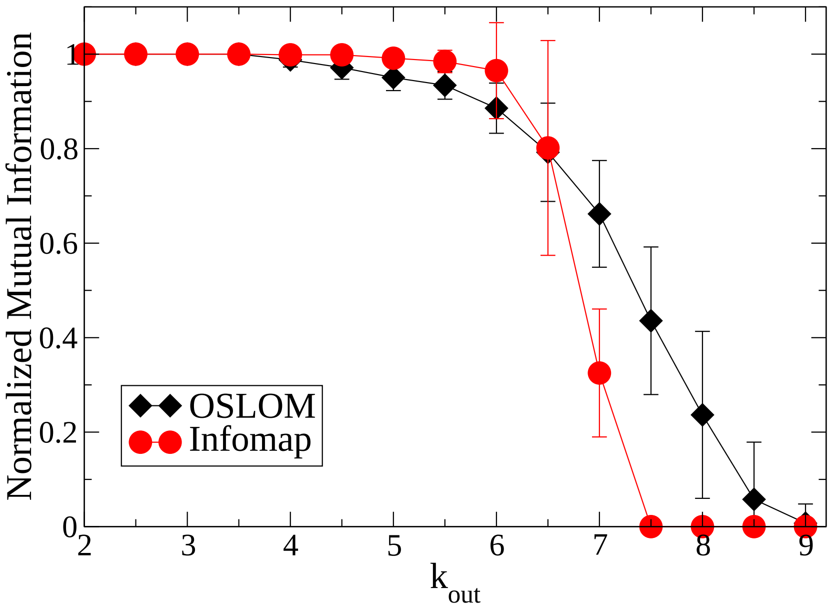

Figure S1. Test on the Girvan-Newman benchmark graphs. The variable k_{out} is the average number of external neighbors per vertex. The two curves refer to OSLOM (diamonds) and Infomap (circles).

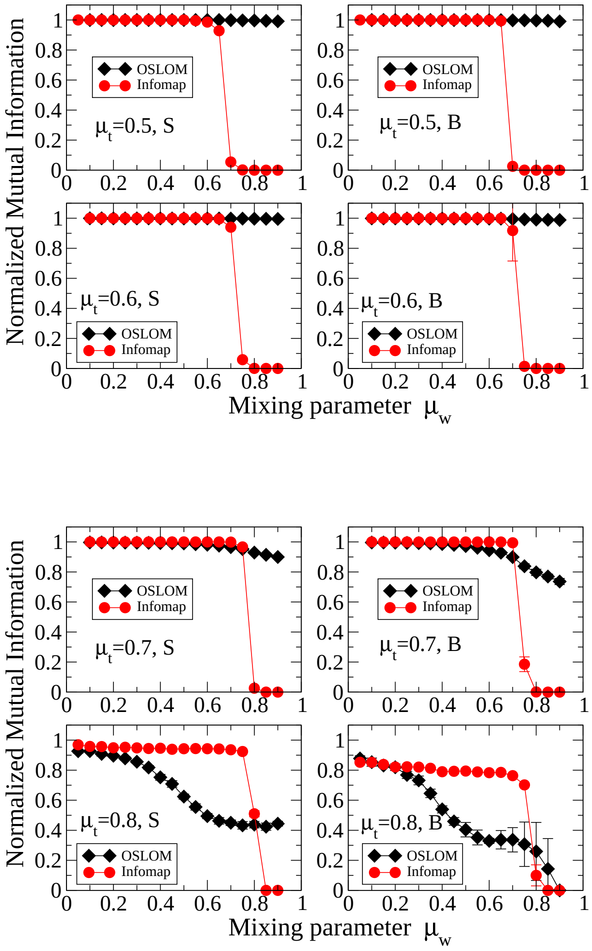

Figure S2. Test on weighted LFR benchmark graphs (undirected and without overlapping communities). The parameters are: $N = 5000$, $\langle k \rangle = 20$, $k_{max} = 50$, $\tau_1 = 2$, $\tau_2 = 1$, $\beta = 1.5$. Each panel corresponds to a given value of the topological mixing parameter μ_t and of the community range (S or B).

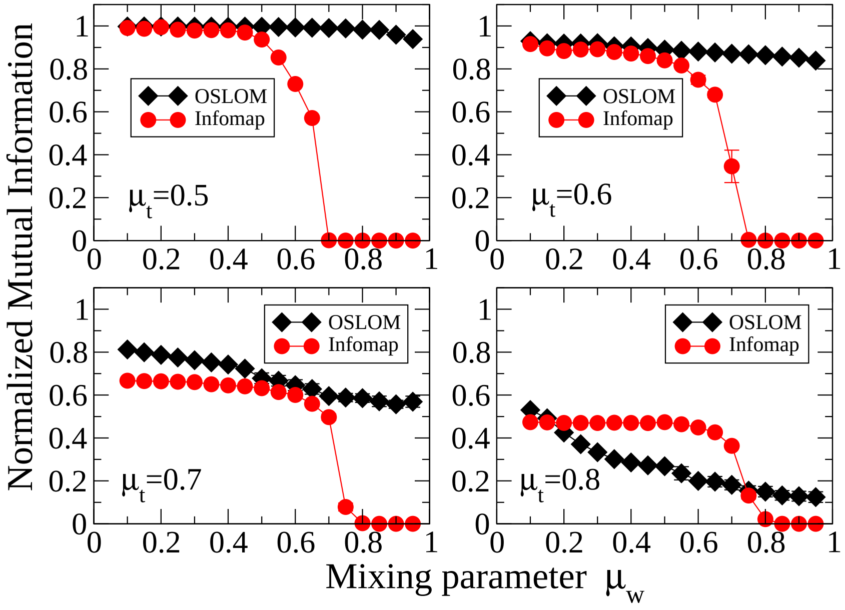

Figure S3. Test on weighted LFR benchmark graphs (undirected and without overlapping communities). The parameters are: $N = 50000$, $\langle k \rangle = 20$, $k_{max} = 200$, $\tau_1 = 2$, $\tau_2 = 1$, $\beta = 1.5$. Each panel corresponds to a given value of the topological mixing parameter μ_t. The range of community sizes is $[20, 1000]$.

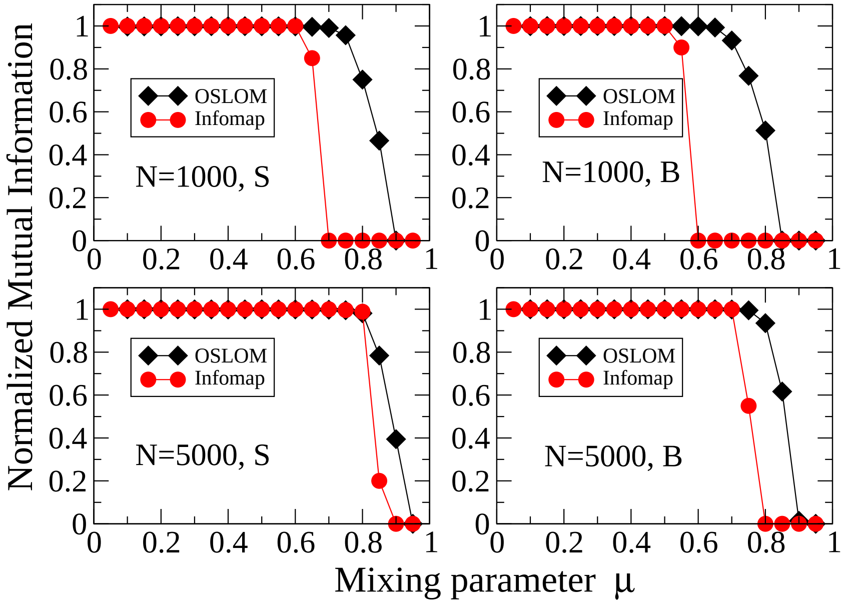

Figure S4. Test on directed LFR benchmark graphs (unweighted and without overlapping communities). The parameters are: $\langle k \rangle = 20$, $k_{max} = 50$, $\tau_{in} = 2$, $\tau_2 = 1$. Each panel corresponds to a given network size ($N = 1000, 5000$) and community range (S or B). The mixing parameter μ refers to in-degree.

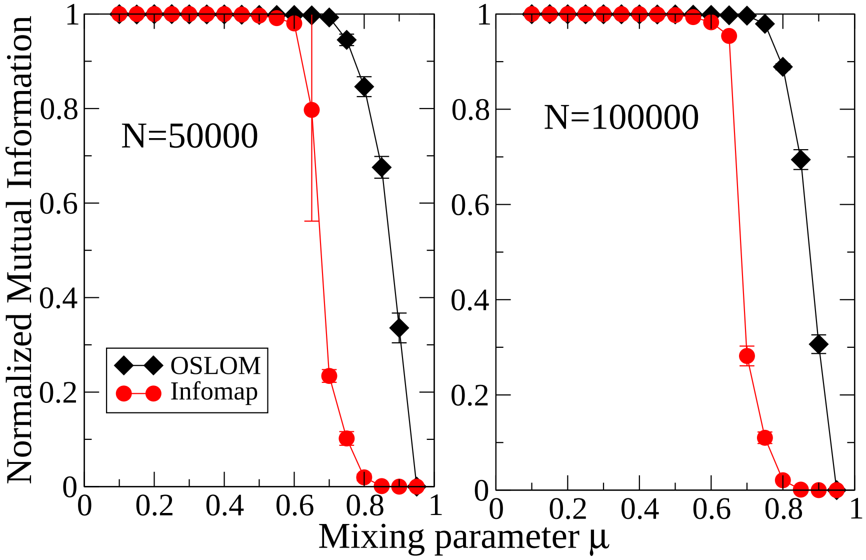

Figure S5. Test on directed LFR benchmark graphs (unweighted and without overlapping communities). The parameters are: $\langle k \rangle = 20$, $k_{max} = 200$, $\tau_{in} = 2$, $\tau_2 = 1$. We consider two large network sizes: $N = 50000$ (left) and $N = 100000$ (right). The range of community sizes is $[20, 1000]$. The mixing parameter μ refers to in-degree.

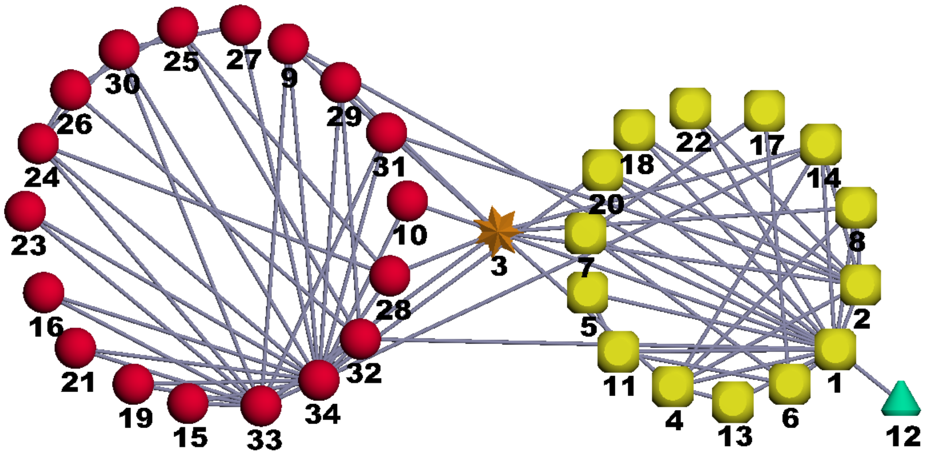

Figure S6. Application of OSLOM to real networks: Zachary's karate club.

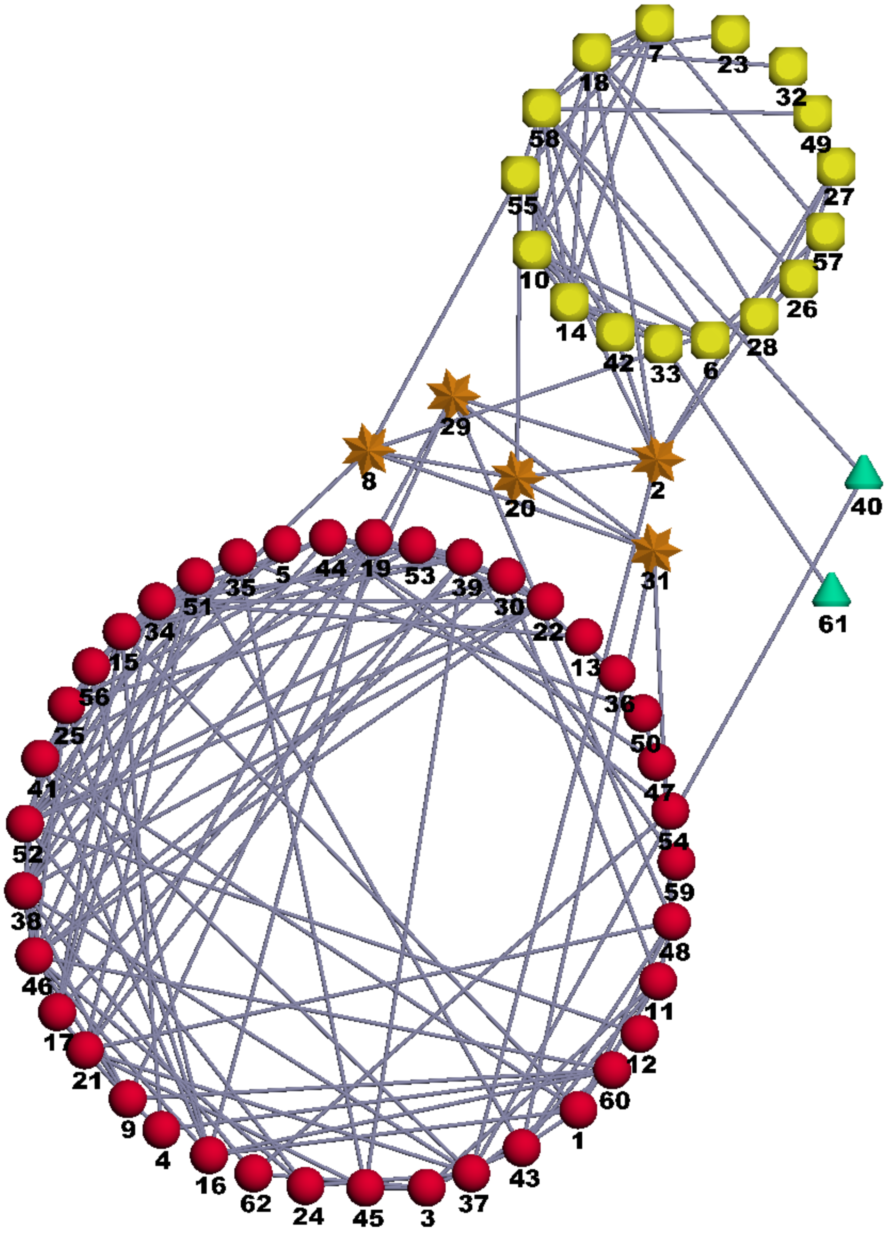

Figure S7. Application of OSLOM to real networks: Lusseau's social network of bottlenose dolphins.

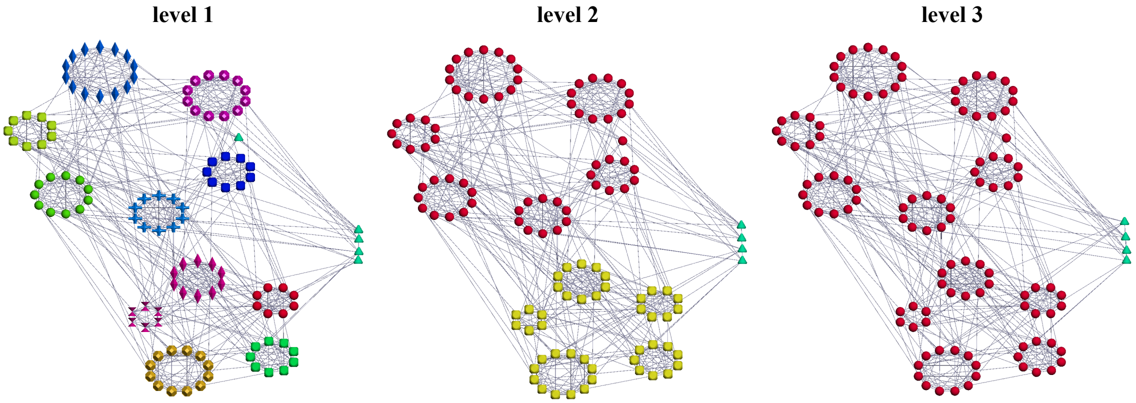

Figure S8. Application of OSLOM to real networks: American college football network.

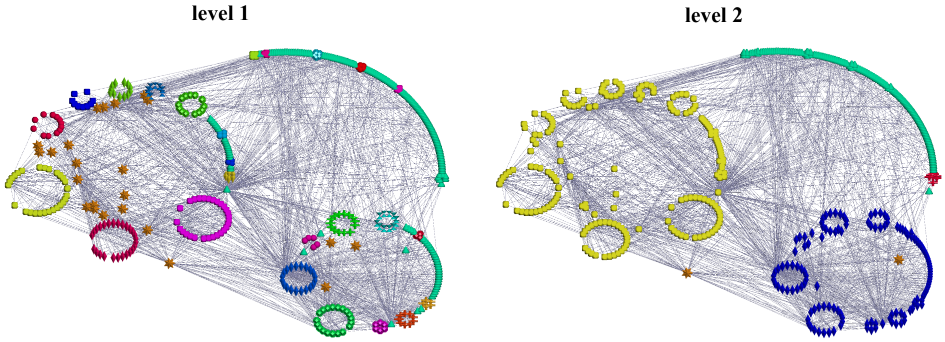

Figure S9. Application of OSLOM to real networks: metabolic network of *C. elegans*.
